# Neural Networks as Cognitive Models of the Processing of Syntactic Constraints

**DOI:** 10.1162/opmi_a_00137

**Published:** 2024-05-06

**Authors:** Suhas Arehalli, Tal Linzen

**Affiliations:** Department of Mathematics, Statistics, and Computer Science, Macalester College, Saint Paul, MN, USA; Department of Linguistics and Center for Data Science, New York University, New York, NY, USA

**Keywords:** computational modeling, neural networks, agreement attraction, syntactic processing, psycholinguistics

## Abstract

Languages are governed by *syntactic constraints*—structural rules that determine which sentences are grammatical in the language. In English, one such constraint is *subject-verb agreement*, which dictates that the number of a verb must match the number of its corresponding subject: “the dog*s* run”, but “the dog run*s*”. While this constraint appears to be simple, in practice speakers make agreement errors, particularly when a noun phrase near the verb differs in number from the subject (for example, a speaker might produce the ungrammatical sentence “the key to the cabinets are rusty”). This phenomenon, referred to as *agreement attraction*, is sensitive to a wide range of properties of the sentence; no single existing model is able to generate predictions for the wide variety of materials studied in the human experimental literature. We explore the viability of neural network language models—broad-coverage systems trained to predict the next word in a corpus—as a framework for addressing this limitation. We analyze the agreement errors made by Long Short-Term Memory (LSTM) networks and compare them to those of humans. The models successfully simulate certain results, such as the so-called number asymmetry and the difference between attraction strength in grammatical and ungrammatical sentences, but failed to simulate others, such as the effect of syntactic distance or notional (conceptual) number. We further evaluate networks trained with explicit syntactic supervision, and find that this form of supervision does not always lead to more human-like syntactic behavior. Finally, we show that the corpus used to train a network significantly affects the pattern of agreement errors produced by the network, and discuss the strengths and limitations of neural networks as a tool for understanding human syntactic processing.

## INTRODUCTION

Every language is governed by a set of *syntactic constraints*—rules that determine whether a particular sentence is acceptable in that language. These rules are often independent of the meaning of the sentence: although most listeners would be able to interpret either “the dog **is** running” and “the dog **are** running” as referring to a running dog, only “the dog **is** running” is a grammatical English sentence. A core goal of psycholinguistics is to determine how such syntactic constraints are enforced in real-time sentence production and comprehension.

Amongst those syntactic constraints, *agreement* is both simple and extraordinarily widespread. Put simply, an agreement constraint requires that two or more syntactic elements share a particular set of features. Most varieties of English exhibit *subject-verb number agreement*, where subject noun phrases and their corresponding verbs must share their number feature: they must either both be singular, or both be plural (e.g., “the dog run*s*,” but “the dog*s* run”).

While this constraint is simple to state, speakers sometimes fail to apply it correctly. Subject-verb agreement errors are particularly likely to arise in sentences with an *attractor*: a noun phrase with a number feature different than that of the subject (e.g., the attractor “cabinets” might give rise to the erroneous “The key to the cabinets **are** rusty”; Bock & Miller, 1991). These errors occur in both production and comprehension (Bock & Miller, 1991; Pearlmutter et al., 1999), and are modulated by a number of factors, including, among others, the type of syntactic constituent the attractor appears in (Bock & Cutting, 1992) and the linear or syntactic distance from the attractor to the verb (Franck et al., 2002; Haskell & MacDonald, 2005; Vigliocco & Nicol, 1998).

A complete theory of language comprehension and production must provide an account of how syntactic constraints are enforced during processing and of the ways in which the computations enforcing those constraints fail. While many proposals for such an account of agreement mechanisms exist in the literature—Marking and Morphing (Eberhard et al., 2005), Retrieval Interference (Badecker & Kuminiak, 2007; Wagers et al., 2009, etc.), and Feature Percolation (Franck et al., 2002, etc.), among others—few proposals can account for the full empirical picture. These accounts typically focus on a particular agreement phenomenon, and do not attempt to be fully specified with respect to the wide array of other agreement phenomena documented in the literature. For example, it is unclear how retrieval interference accounts would predict notional number effects (Humphreys & Bock, 2005), and underspecification in parts of the model—for instance, the choice of retrieval cues available—makes it difficult to ascertain whether this reflects a failure on the part of the account or a justification for a different set of cues to handle this particular situation.

The goal of this paper is to work towards an alternative approach to constructing such a comprehensive account of agreement processing. We leverage the success of the broad-coverage neural network language models—that is, word prediction models—that are widely used in applied language technologies. These language models are designed to take as input a sequence of words and predict the following word in that sequence. They are typically trained on a large corpus of naturally occurring text, which allows them to learn any number of syntactic or semantic properties from their training data. They are provided no explicit supervision, and as such will only learn properties of the language that are helpful for their training task: word prediction. We adopt these models for two reasons. First, unlike previous models of agreement attraction, they are *broad-coverage*: they can take as input any sequence of words and generate predictions for the next word. Second, neural network language models have been shown to be generally capable of enforcing subject-verb agreement in English, while making occasional agreement errors (Gulordava et al., 2018; Linzen et al., 2016). Taken together, these properties allow us to efficiently derive agreement predictions from the models for any set of sentences and compare the errors in those predictions to those made by humans.

Unlike traditional cognitive models, which explicitly implement the mechanisms that researchers hypothesize are used by humans, processing mechanisms in neural language models emerge naturally over the course of training. As a result, it is much more difficult to describe in words the precise cognitive mechanism a neural network model implements. Rather than interpret the exact mechanisms that govern a neural network model’s behavior, it is often useful to understand the model in terms of the pressures that influence the kinds of representations and mechanisms the model can learn. The processing mechanisms the model develops over the course of training are the product of two factors: first, the model’s inductive biases, or the factors that lead a model to generalize in particular ways from its finite training data (e.g., architecture, or optimization procedure); and second, the training data and task. As such, characterizing the effect of these components on the outcome of learning serves as a way of understanding the mechanism the model implements (i.e., a reasonable hypothesis is that the model will implement the mechanism that is optimal to learn under the constraints of architecture and task).

This suggests a paradigm through which we can characterize potential mechanisms underlying language processing behavior: manipulate a neural language model’s architecture or training objective(s), and compare the behavior of those models to that of humans. By characterizing the manipulations that result in models producing human-like behavior, we can gain insight into the conditions under which human-like language processing can arise: do particular learning pressures make human language processing strategies optimal? Does a pressure toward a particular representational structure in addition to a word prediction objective make human error patterns emerge? Can we derive complex behavioral results from an interaction of simple biases and learning pressures?

We adopt this approach to investigate whether pressure towards learning a particular, linguistically motivated structural representation align neural network models more closely with human behavior. We evaluate two types of models based on the Long-Short Term Memory (LSTM) neural network architecture (Hochreiter & Schmidhuber, 1997): models trained solely to predict the next word, and models trained to predict the next word and also labels from the Combinatory Categorial Grammar (CCG) syntactic formalism. We derive predictions from each of the two types of models for six sets of findings from the human agreement processing literature. Both sets of models successfully simulated a number of empirical findings, but failed to simulate others. Adding the explicit syntactic training objective had mixed results: in some cases it aligned the models’ error patterns more closely with those of humans, but in other cases it did not. We conduct follow-up analyses which suggest that even more sophisticated syntactic pressures may be necessary to bring models closer to human behavior.

We then consider the other major kind of learning pressure: the training data. In our main experiments, models were trained on a concatenation of a subset of English Wikipedia and the CCGBank corpus of news articles (Hockenmaier & Steedman, 2007). We conduct follow-up experiments where we trained models either solely on the Wikipedia subset or solely on CCGBank. We found that both the size and genre of the training corpus affected the errors the models made. We take this to suggest that (1) neural network language models used as cognitive models may need to incorporate stronger inductive biases, not only to encourage more human-like behavior, but also to reduce sensitivity to the composition of their training corpora; and (2) researchers working on cognitive modeling with language models should aim to train those models on corpora that accurately reflect the data humans learn from.

All of our LSTM models, which were trained on small to moderately-sized corpora by the standard of the language technologies world, displayed larger overall error rates than humans. This raises two questions: first, whether this is an issue with neural network models broadly, or if it is just the result of the scale and architecture of the models we’ve chosen. Second, whether aiming simply to reduce this error rate (by, for instance, training more powerful models) will give us the human-like error patterns we are interested in. To address these questions, we conducted additional follow-up simulations using the publicly available GPT-2 language model (Radford et al., 2019), which was trained on many billions of words and is based on the Transformer neural network architecture (Vaswani et al., 2017). We found that, though GPT-2 displays a lower overall error rate, this overall improvement does not translate into a more human-like error pattern.

Before we describe our simulations in detail, we provide a brief introduction to agreement and agreement attraction in English, and discuss related prior work modeling human language processing with neural language models and how the present work fits into this landscape.

### Subject-Verb Agreement and Agreement Attraction in English

Subject-Verb agreement is a constraint in many dialects of English that requires the number feature of a subject to match the number of the corresponding verb, as in Example 1. A mismatch in number features results in the ungrammatical Example 2.

(1)  The key opens the door.
(2)  *The key open the door.

This constraint holds regardless of what noun phrases (NPs) appear elsewhere in the sentence, as shown in Example 3 and Example 4.

(3)  The key to the cabinet opens/*open the door.
(4)  The key to the cabinets opens/*open the door.

In practice, human behavior can deviate from this description. Agreement errors occur occasionally in many contexts, and are particularly common in the presence of an NP whose number feature does not match that of the subject, such as Example 4: in this example, a higher error rate is expected compared to the minimally different Example 3 (Bock & Miller, 1991).

This pattern of errors was originally documented in the sentence completion paradigm. In this paradigm, participants are given a prefix of a sentence up to but not including the main verb, as in Example 5 or 6, and are tasked with completing the sentence:

(5)  The key to the cabinets …
(6)  The key to the cabinet …

The experimenter then determines if the participant produced a grammatical verb that matches the number of the subject, like *is*, or an ungrammatical verb, like *are*. Following Bock and Miller’s (1991) study, agreement attraction has also been documented in comprehension (Parker & An, 2018; Pearlmutter et al., 1999; Wagers et al., 2009), and similar findings have been reported across languages (Franck et al., 2002, 2006; Lorimor et al., 2008, among others).

The magnitude of the agreement attraction effect—the difference in error rates between Example 5 and 6, for example—is sensitive to a variety of factors, both syntactic (Bock & Cutting, 1992; Franck et al., 2002, etc.) and semantic (Humphreys & Bock, 2005; Parker & An, 2018, etc.). A number of theories have been proposed to explain the influence of these factors on agreement; these include the Marking & Morphing model (Eberhard et al., 2005, etc.), feature percolation accounts (Franck et al., 2002, etc.), and memory retrieval-based accounts (Wagers et al., 2009, etc.). Each account is motivated by a particular subset of the empirical findings that are best explained by that account: notional number effects motivate the Marking & Morphing model (Humphreys & Bock, 2005, etc.), syntactic distance effects motivate feature percolation accounts (Bock & Cutting, 1992; Franck et al., 2002, etc.), and linear distance effects (e.g., Haskell & MacDonald, 2005) and grammaticality asymmetry effects (Wagers et al., 2009) motivate memory retrieval-based models.

In this paper, we use neural networks to simulate six human experiments that span the three groups of results that have motivated previous accounts. The findings of these experiments can be summarized as follows: (1) attractors in prepositional phrases give rise to a stronger attraction effect than those in relative clauses, and plural attractors generate a stronger attraction effect than singular attractors (Bock & Cutting, 1992); (2–3) attractors closer to the verb exert a stronger attraction effect, whether distance is measured in syntactic (Franck et al., 2002) or linear (Haskell & MacDonald, 2005) terms; (4) collective subjects with distributive readings have higher rates of plural agreement than those with collective readings (Humphreys & Bock, 2005); (5) attractors in oblique arguments cause a larger attraction effect than those in core arguments (Parker & An, 2018); and (6) attraction can be caused by attractors outside of the clause containing the agreement dependency, and while attraction makes ungrammatical sentences seem grammatical, it does not make grammatical sentences seem ungrammatical (Wagers et al., 2009).

### Subject-Verb Agreement in Neural Language Models

Most relevant prior work on neural language models has evaluated the extent to which neural networks obey grammatical agreement constraints, and was not directly concerned with comparing the networks’ errors to those made by humans. Elman (1991) evaluated Simple Recurrent Networks (SRNs) trained to predict the next word in a small artificial corpus and found that the models were capable of predicting the number of verbs accurately, even when the subject and verb were separated by a relative clause. More recently, Linzen et al. (2016) trained Long-Short Term Memory models (LSTMs) using a number of objectives, including word prediction, and evaluated whether they predicted the correct number inflection of the verb on preambles extracted from Wikipedia, which include naturally occurring attractors. While they concluded that word prediction alone was insufficient to learn agreement dependencies from natural corpora, Gulordava et al. (2018) later reached a different conclusion, demonstrating that a better trained LSTM language model could successfully learn agreement dependencies through word prediction, even when evaluated on so-called “colorless green ideas” preambles that are stripped of any semantic content that could facilitate agreement processing. Agreement across simple intervening noun phrases has also been a consistent part of syntactic benchmarks for language models (Hu et al., 2020; Marvin & Linzen, 2018; Warstadt et al., 2019, 2020), with modern models performing reasonably well, though with some errors.

Taken together, this body of work provides robust evidence that neural network language models are capable of representing subject-verb number agreement dependencies, though these representations have their limitations. Yet it is much less clear *what* representations those models employ for agreement dependencies, and how robust those representations are. One line of work aiming to address this question for RNNs has found evidence for a single pair of singular and plural units per model that represent number information for all subject-verb relationships within a sentence (Lakretz et al., 2019, 2021). Another line of work analyzing Transformer models (Vaswani et al., 2017), such as GPT-2 (Radford et al., 2019), suggests that attraction effects may be the result of the transformer’s attention mechanism being subject to the same sorts of similarity-based interference effects as cue-based models from the human memory literature (Ryu & Lewis, 2021).

As mentioned above, most prior work has not compared the neural networks’ detailed error patterns to those of humans. One exception is Linzen and Leonard (2018), who found that the models they trained exhibited agreement attraction errors, in general, as well as number asymmetry effects (with plural noun phrases exerting a stronger attraction effects than singular ones), but did not show higher error rates with attractors in prepositional phrases than with attractors in relative clauses (as was found for humans by Bock & Cutting, 1992). However, the models used by Linzen and Leonard (2018) were not word prediction models, but classifiers trained solely to predict the number feature of the verb. This modeling setting is difficult to compare to the rest of the literature, which is concerned with word prediction models. This objective is also less cognitively plausible: unlike the classifier, which is focused only on verb number prediction, humans need to learn and process all aspects of language at the same time, and are not provided with explicit supervision about verb number.

Like Linzen and Leonard (2018), the current work aims to model the patterns of agreement errors that humans produce. Unlike in their work, however, we use models trained on the general, broad-coverage word prediction task, rather than models tailor-made for agreement prediction. This requires us to use linking functions that relate the models’ probability distribution over the upcoming word to human behavioral measures. We discuss these linking hypotheses, as well as our modeling and statistical choices, in detail in the next section.

The goals of this work are distinct from but related to a line of work investigating the inductive biases or types of training data necessary for models to acquire human-like syntactic capabilities (McCoy, Frank, & Linzen, 2020; Wilcox et al., 2018, 2023; Yedetore et al., 2023, etc.). While we are motivated by the fact that the language processing strategies acquired by neural network are inherently learnable (which is not necessarily the case for all other cognitive models), in this work our primary goal is modeling syntactic behavior in adults, rather than modeling acquisition. This is most clearly seen in our use of an auxiliary syntactic training objective to pressure our models to learn syntactic representations. We make no claims that the training signal provided by this task is used in the same way during human language acquisition; instead, we use this task to test the hypothesis that representations equivalent to those learned by training on this task lead models to more human-like behavior. Another distinction between these lines of work and ours lies in the kinds of data they seek to explain. Both Wilcox et al. (2023) and the current work compare the syntactic abilities of humans and neural networks. But we are primarily focused on modeling where human syntactic processing *fails*, and what those errors reveal about human processing mechanisms, while Wilcox et al. (2023), Yedetore et al. (2023), etc. are interested in syntactic phenomena that humans are largely successful at but are purported to be difficult for simple neural models to learn (i.e., challenging versions of the poverty of the stimulus argument; Chomsky, 1965, 1986).

## METHODS

### Language Models

Language models are natural language processing systems that assign probabilities to strings of words in a language. In this work, we focus on autoregressive language models—models that decompose the task of assigning probability to a sequence of words into the simpler task of providing a probability distribution over the next word in a sequence given all prior words (i.e., “predicting the next word word in a sequence”).^1^ We primarily use language models based on the LSTM architecture, a type of *Recurrent Neural Network* (RNN) architecture. We briefly describe this neural network architecture in the remainder of this section.

RNNs transform a sequence of vector representations (representing, for example, words in a sentence) into a single vector representation by iteratively merging a vector representation of the left context (*h*_*i*−1_) with a vector representation of the input to the right of that context (*w*_*i*_) until all of the vectors are merged. In Simple Recurrent Networks (SRNs, Elman, 1990), vectors are merged using Equation 1. The weight matrices *W*_*h*_ and *W*_*w*_ are learned linear transformations that are applied to *h*_*i*−1_ and *w*_*i*_ respectively; the outcomes are summed and transformed by a non-linear activation function (in this case, the hyperbolic tangent function):

$$h_{i}=\tanh\left(W_{h}h_{i-1}+W_{w}w_{i}\right)$$ (1)

**Figure 1.**
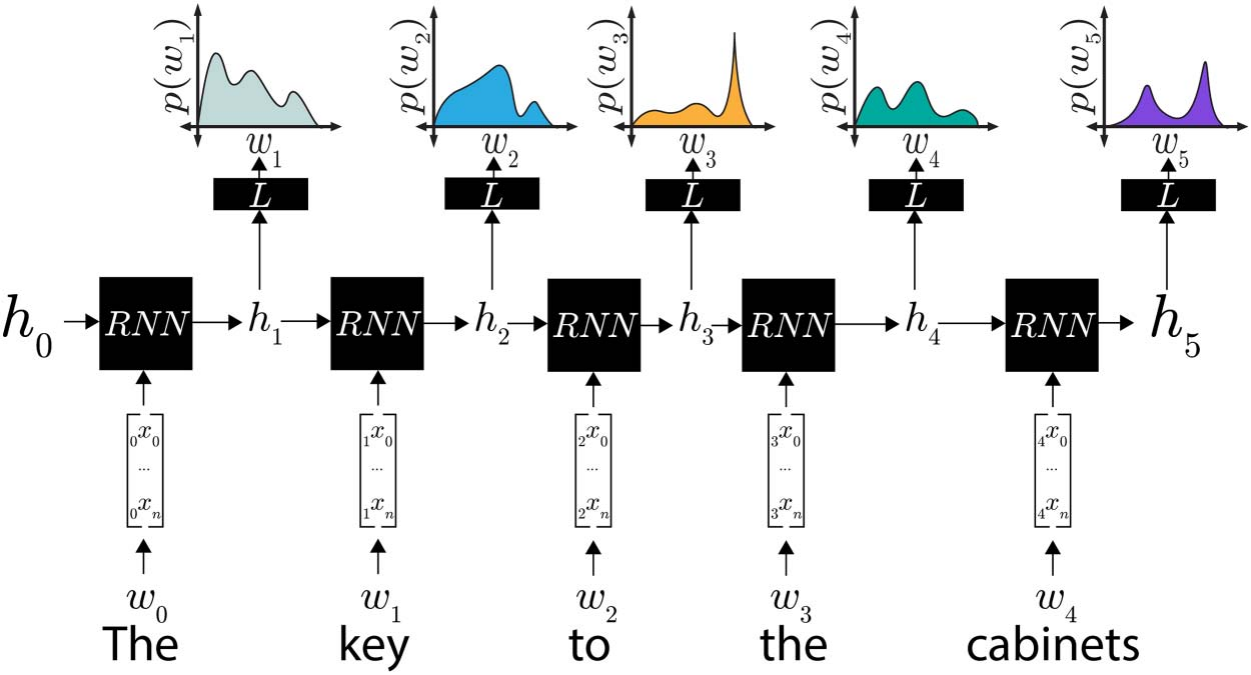
In our language modeling setup, each word is mapped to a word vector. Each of those representations is combined with a representation of all previous words (*h*_*i*−1_) using a recurrent neural network model (*RNN*) to create a representation *h*_*i*_ for all words up to word *i*. To generate a prediction for word *i*, *h*_*i*_ is fed into a linear decoder (*L*) to generate a distribution over word *i*. During training, model weights (which determine *RNN* and *L*) are adjusted to maximize the probability of the word that actually occurred in the sentence at position *i*.

In a neural network language model, words from the training data are mapped to learned vector embeddings, and sequences of those embeddings are fed into a neural network encoder that, like the recurrent network described above, produces a single vector that represents that sequence of words. That representation is then provided as the input to a linear decoder—a learned linear transformation followed by a softmax operation—which outputs a probability distribution over the model’s vocabulary (see Figure 1). The model’s task is to align this probability distribution with the empirical probability that any particular word in the model’s vocabulary is the next word in the sequence. Before training, all of the model’s learned weights—in a simple recurrent network, those are the embedding mappings, the two weight matrices *W*_*h*_ and *W*_*w*_, and the matrix representing the linear transformation in the encoder—are randomly initialized, and so the model’s output probability distribution is essentially random. For each training example, all of those weights are adjusted using stochastic gradient descent so as to increase the likelihood of the true next word from the training data.

Our simulations primarily use LSTMs, a type of RNN that incorporates gating mechanisms designed to maintain representations over longer sequences; these mechanisms mitigate the issue that, due to successive merging operations, representations derived from early words have little effect by the end of the sequence. These gating mechanisms yield better representations of long-distance dependencies (Bhatt et al., 2020), which makes them better suited than SRNs for modeling agreement relations, and, in turn, agreement attraction. On a conceptual level, however, LSTMs fundamentally operate by the same principles as SRNs: they incrementally merge inputs from left to right using a trainable, parametrized function.

In order to evaluate whether more sophisticated model architectures and training regimes can address issues of high error rates found in our LSTM-based models, we additionally consider GPT-2 (Radford et al., 2019), a language model based on the Transformer architecture (Vaswani et al., 2017). Unlike the RNN models described above, Transformer language models do not predict the next word from a representation generated by an incremental left-to-right composition operation. Instead, they construct representations using a mechanism called *self-attention*, where the model has direct access to representations of prior words. GPT-2 differs from our LSTMs in many dimensions, and thus direct comparisons between the models are difficult. However, since Transformer models like GPT-2 have had great success recently (including in modeling psycholinguistic data, e.g., Oh et al., 2022; Schrimpf et al., 2021), we provide results for GPT-2 not as a part of any direct manipulation, but as an indicator of how larger, more powerful language models fare in their ability to match human agreement error behavior. To preview the results of our experiments, we find that GPT-2 models do perform better than LSTMs syntactically (i.e., they assign greater probability to grammatical forms), but their errors do not uniformly pattern more like human errors than LSTM errors do.

### Model Architectures and Training Setup

**Figure 2.**
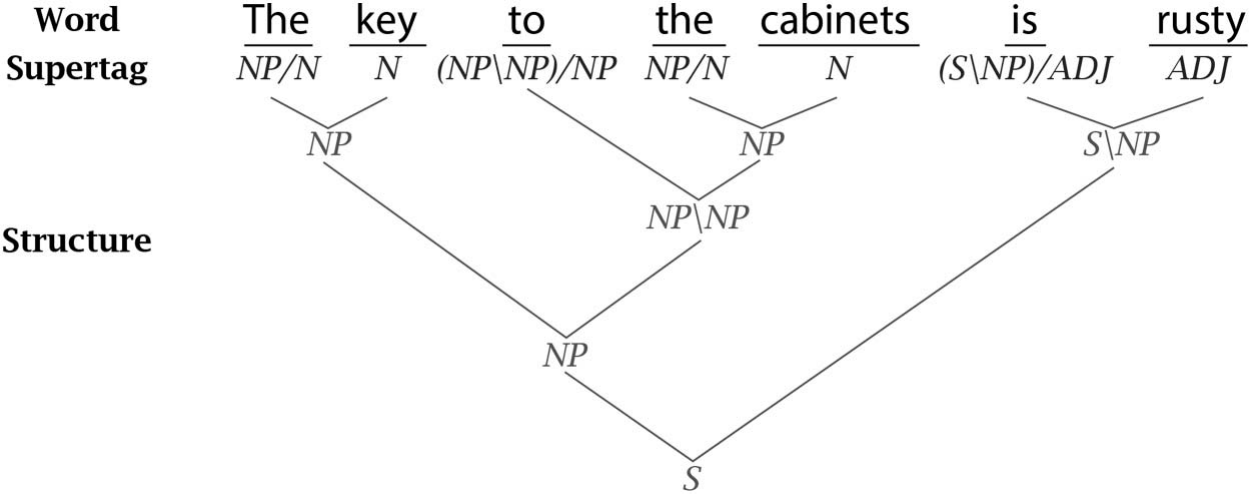
An example sequence of CCG supertags for the sentence *The key to the cabinets is rusty*. Each supertag encodes how the corresponding word composes with its syntactic neighborhood. The label *Y*/*X* denotes that the word it labels merges with a constituent of type X on its right to form a constituent of type Y (as with *the* and *key*), and *Y*\*X* denotes the same, but with the constituent of type X on its left (as with *to the cabinets* and *the key*). To predict supertags successfully, models must learn to represent something akin to the underlying structure of the sentence. In many cases, knowing the sequence of supertags makes it possible to deterministically reconstruct the full parse of the sentence.

For each of the six human experiments we discuss, we compare human behavior to simulation results from the publicly available GPT-2 model, as well as two types of LSTM-based models we train—models trained only on word prediction (LM-Only models) and multi-task models, which are trained on both word prediction and *Combinatory Categorial Grammar Supertagging* (LM+CCG; Steedman, 1987). The multi-task models are trained to predict, from a sequence of words, not only the next word, but also the most recent word’s *supertag*—an enriched part-of-speech tag that encodes local syntactic information (see Figure 2). Due to the rich syntactic information contained in supertags, supertagging has been described as “almost parsing” (Bangalore & Joshi, 1999), and so we hypothesize that jointly optimizing for both supertagging and language modeling accuracy will imbue a model with an additional bias toward learning more sophisticated syntactic representations (Enguehard et al., 2017; Qian et al., 2021).

We trained five instances of each model. The weights of each of these instances was randomly initialized separately; training multiple model instances with different initial weights allows us to determine to what extent the behavior observed is dependent on particular initial weights (McCoy, Min, & Linzen, 2020), much like group-level analyses in psychology. The five LM-Only model instances were trained for 12 epochs over the 80 million words of English Wikipedia used in Gulordava et al. (2018), concatenated with the approximately one million words of the Wall Street Journal section of the Penn Treebank (WSJ Corpus; Marcus et al., 1993). Following Gulordava et al. (2018), the RNN encoder in each model was a 2-layer LSTM with 650 hidden units in each layer. LM-Only models achieved perplexities between 66.73 and 67.13 over the Wikipedia corpus’ test set.^2^

**Figure 3.**
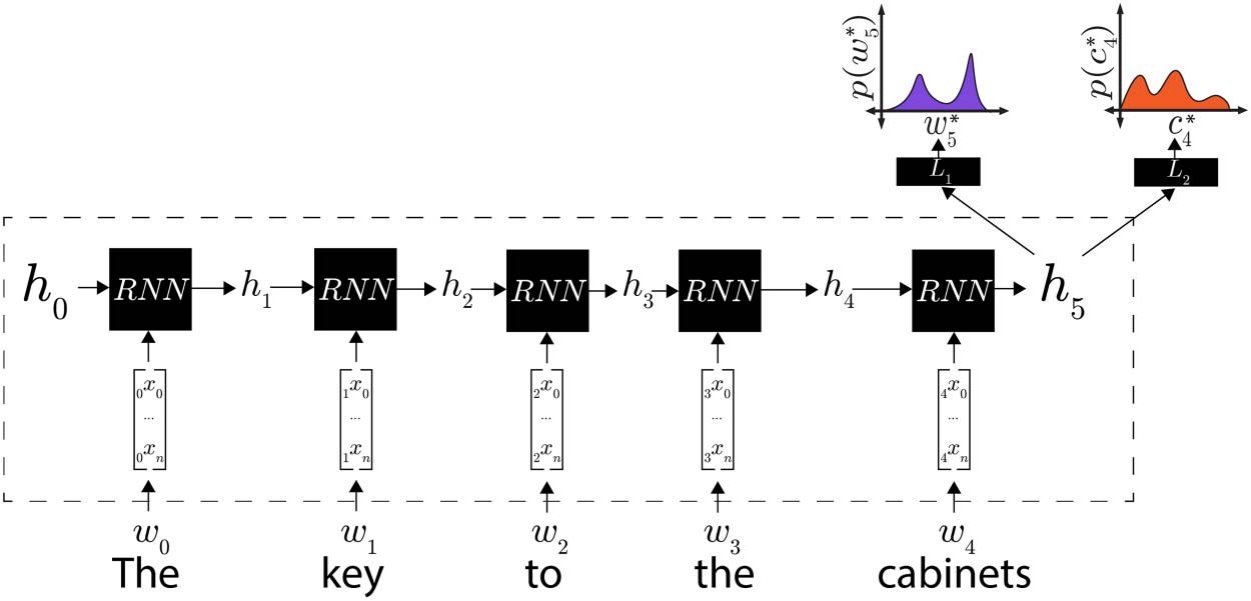
An outline of the architecture used for the LM+CCG models. Using the internal representation *h*_5_ constructed by an RNN encoder, classifier *L*_1_ generates a probability distribution over possible next words w* and classifier *L*_2_ generates a probability distribution over possible supertags c* for the current word.

The five LM+CCG model instances were trained on both word prediction and supertagging: in addition to the linear decoder that predicted the next word, a secondary linear decoder predicted the current word’s supertag. The structure of this multi-classifier architecture is outlined in Figure 3. Word prediction was performed over the 80 million words taken from English Wikipedia (Gulordava et al., 2018), supplemented with approximately one million words of the WSJ Corpus. CCG supertagging was performed over CCGbank (Hockenmaier & Steedman, 2007), a version of the WSJ Corpus annotated with CCG derivations. The two training objectives—word prediction and supertagging—were weighted equally in training. LM+CCG models achieved language modeling perplexities ranging from 74.76 to 75.70 on the Wikipedia test set, and assigned the highest likelihood to the correct CCG supertag between 84.1% and 84.5% of the time. This is substantially higher than the accuracy of a baseline that selects the most frequent supertag for each word independent of its context, which is 71.2% (Clark, 2002); this suggests that the models have learned a considerable amount about local syntactic structure, and thus lends credence to our belief that our supertagging models learn relatively sophisticated syntactic representations.

The models described so far were trained on the concatenation of two distinct corpora that differ in both size and genre. Given the sharp differences between these two corpora, we also trained two additional sets of models with the LM-Only architecture on each of those corpora in order to determine whether a particular size or writing style affected the models’ agreement behavior. Five model instances were trained on the 80 million word Wikipedia corpus, and five were trained on the approximately one million words of the WSJ Corpus. Test-set perplexities for models trained on Wikipedia data ranged between 67.66 and 68.15, and those for models trained on WSJ data ranged between 55.32 and 56.13.

Finally, our GPT-2 simulations employed the “small” 124 million parameter GPT-2 model (Radford et al., 2019), trained on roughly 40GB of text scraped from the internet. This model achieves a perplexity of 65.85 over the WSJ Corpus. We remind the reader that due to differences in tokenization and test sets, perplexities in this sections are not directly comparable.

### Linking Model Outputs to Human Behavior

The behavioral data in the experiments we simulate has one of two forms: the proportion of singular verbs produced in a sentence completion paradigm, or the reading time of words in a critical region in a self-paced reading study. Both paradigms are discussed in more detail in this section. As we described in the prior sections, a language model takes as input a sequence of words and outputs a probability distribution over the next word in that sequence. To compare the performance of these models to that of humans, we need to link the language model’s output to the behavioral responses recorded in the human experiments. This section discusses how we select an appropriate linking function, and how we combine it with a language model to construct what we will, in future sections, refer to simply as our (cognitive) model.^3^

#### Predicting Reading Times.

The comprehension studies we simulate have employed the self-paced reading paradigm. In self-paced reading, participants are presented with sentences one word at a time; the next word is revealed after the participant presses a particular button. The dependent measure is the time that elapses between two key presses (the displayed word’s *reading time*). Longer reading times are taken to indicate greater processing difficulty caused by the word currently being displayed, or by one of the words immediately preceding it.

In the context of agreement processing, reading times at the verb can indicate how acceptable the participant finds the subject-verb agreement relation in question. The logic of this paradigm relies on the observation that encountering an agreement violation incurs processing cost, which leads to longer reading times at the verb or at the words immediately after it. Agreement attraction can then surface in one of two manners: the amelioration of an agreement error, where ungrammatical sentences are read faster when an attractor matches the number of the verb, making it harder to detect the error; and the illusion of an agreement error, where grammatical sentences are read slower when an attractor mismatches the number of both the subject and verb (Pearlmutter et al., 1999; Wagers et al., 2009). We will discuss this logic in more detail when we describe the two comprehension experiments we simulate.

In order to convert the probability distributions provided by language models into a measure comparable with reading times, we use *surprisal* (Hale, 2001; Levy, 2008), defined in Equation 2.

$$\text{Surprisal}\left(w_{i}\right)=-\log_{2}\left(P\left(w_{i}|w_{0},\ldots ,w_{i-1}\right)\right)$$ (2)

Note that the probability *P*(*w*_*i*_ | *w*_0_, …, *w*_*i*−1_) is the probability that the *i*th word in the sequence is *w*_*i*_, given that all of the prior words are *w*_0_, …, *w*_*i*−1_. This is precisely the probability distribution we obtain from a language model after it has been given *w*_0_, …, *w*_*i*−1_ as input. The relationship between human reading times and surprisal estimated from a language model in this fashion has been found to be approximately linear (Shain et al., 2024; Smith & Levy, 2013).

#### Predicting Verb Completions.

The production studies we simulate all used the sentence completion paradigm briefly described above. In this paradigm, participants are asked to repeat and complete a given preamble (in this case, a complex noun phrase), and their responses are coded for the number feature of the verb they produce and whether the agreement relation is grammatical. For example, when provided the preamble “The keys to the cabinet”, a participant might respond with “The keys to the cabinet are on the table”, which would be coded as a plural and grammatical response. Agreement attraction manifests as a higher error rate for preambles where the attractor noun’s number mismatches the subject’s number compared to preambles where the numbers of the two nouns match. To simulate such an experiment with language models, we need to convert the output of the language model—a distribution over the next word in the sentence—to the probabilities with which the model would produce a singular or plural verb.

**Figure 4.**
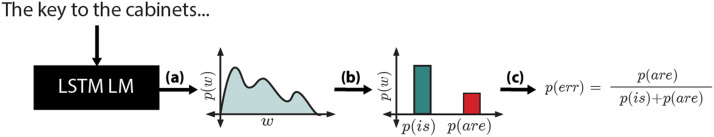
To simulate a sentence completion experiment, a language model is given each preamble as input, producing a probability distribution over the following word (***a***). The probabilities of a candidate singular and plural verb are extracted from this distribution (***b***) and renormalized (***c***) and this new distribution is taken to represent the probability with which the model would produce a singular or plural verb.

For our simulations, we will use what we will refer to as the one-sample linking function. This function is equivalent to having the simulated production process decide on a verb form based on a single sample from the underlying language model’s probability distribution (see the General Discussion for more details and the motivation for the name one-sample). Under this paradigm, we first select a candidate pair of singular and plural forms of a particular verb—for example, *is* and *are*—and compute their probabilities under the distribution provided by the language model. We then renormalize the probabilities over the two candidate words such that they sum to 1, and take the renormalized probabilities as the probabilities with which the model produces a singular or plural verb (see Figure 4).

### Experimental Stimuli

For each simulation, we aimed to use the stimuli provided in the publications that reported on the relevant human experiment. This goal was complicated by the fact that the models can only process words included in their training data; some of the more infrequent words in the experimental stimuli did not occur in the training corpus at all, or were replaced during training with a standard “unknown” (out-of-vocabulary) token (this is standard practice motivated by the fact that language models are unable to learn appropriate vector representations for words that occur a small number of times in the training corpus.) To deal with this issue, we identified any out-of-vocabulary word that was a part of a noun phrase (and thus could potentially contribute number information) or was manipulated in the simulated experiment’s design and replaced it with a semantically similar, in-vocabulary word. Note that this necessarily increases the frequency of the word as estimated using our training corpora, since the original word did not appear in the models’ vocabularies—precisely because it fell under the out-of-vocabulary frequency threshold—while the replacement word did appear in the vocabulary. If the word was not in a noun phrase, or was not relevant to the experimental manipulation, we did not attempt to find a substitute word, and replaced it with the out-of-vocabulary token instead. A summary of the changes we made to the materials can be found in Appendix C.

Due to the limited vocabulary of the models trained on the WSJ Corpus, a larger number of words needed to be adjusted. To avoid editing experimental materials too significantly, we limited our simulations based on these models to the three experiments that focused on syntactic structure: Bock and Cutting (1992), Franck et al. (2002), and Haskell and MacDonald (2005).

The candidate pairs of singular and plural verbs for production experiments were always the present tense forms of the verb *be*. We made this choice this for two reasons: first, these verbs appear with high frequency in the training data, and thus are likely to have number information properly encoded in their vector representations; and second, these verbs are plausible with nearly any subject noun phrase, and thus can be used across a wide variety of stimuli. In Appendix A, we report a simulation of Bock and Cutting (1992) across a wider variety of verbs to demonstrate that our results are largely robust to verb choice.

### Statistical Analysis

For each of our statistical analyses, we first constructed a mixed-effects model with a maximal mixed-effects structure, that is, random slopes and intercepts for each experimental item and model instance. If the statistical model did not converge, the random effects structure was incrementally pruned until convergence was reached. For all mixed-effects models reported below, this procedure resulted in the inclusion of random intercepts only, for both items and model instance.

For the analyses where the response variable was surprisal, we used linear mixed-effects regression. For the analyses where the response variable was a probability, we used beta mixed-effects regression (Ferrari & Cribari-Neto, 2004), which assumes that the dependent variable (the probability of a particular inflection of the verb) is beta distributed. This assumption bounds the value of the dependent variable between 0 and 1, as is appropriate for a probability. To test the significance of each fixed effect, we report the result of either a Wald test (for beta mixed-effects models) or a t-test (for linear mixed-effects models). To test whether two fixed effects are significantly different from each other, we report the results of a linear hypothesis test where we compare the fit of the original mixed-effects model to a model where the two fixed effects in question are constrained to be equal.

## SIMULATIONS

This section describes the results of simulations of the six experiments from the human literature that we examine in this paper. For each experiment, we lay out the motivation and design of the experiment, describe the outcome of the human experiment, and report the results of our simulations. In the Summary of Results section, we synthesize the results of the simulations with respect to the three empirical questions we seek to answer: (1) what agreement phenomena do LM-Only language models capture? (2) what effect does the addition of an explicit syntactic training objective have on a model’s agreement behavior? and (3) how does a model’s agreement behavior depend on the corpus used to train the model?

### Attractors in Prepositional Phrase vs. Relative Clauses

#### Background.

The first three experiments we simulate investigate how hierarchical syntactic structure affects agreement attraction. We first simulate Experiment 1 of Bock and Cutting (1992), in which the authors tested whether attractors located within prepositional phrases (PPs, Examples 7–8) exerted a stronger attraction effects than attractors within relative clauses (RCs, Examples 9–10):

(7)  The demo tape from the popular rock singer …
(8)  The demo tape from the popular rock singers …
(9)  The demo tape that promoted the rock singer …
(10)  The demo tape that promoted the rock singers …

#### Human Results.

Using the sentence completion paradigm (see Methods for further details), Bock and Cutting (1992) compared the strength of the attraction effect within PPs (the difference in error rates between preambles like Example 7 and 8) to that within RCs (the difference in error rates between Example 9 and 10). They found that attraction was stronger from attractors in PPs than attractors within RCs. They also documented a *number asymmetry*: there were more attraction errors in sentences with singular subjects than in sentences with plural subjects.

#### Simulation Results—Modifier Type.

**Figure 5.**
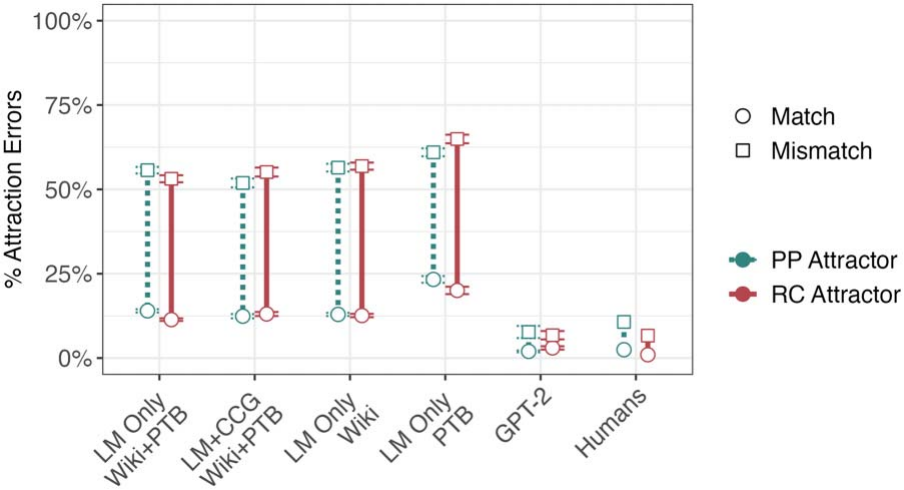
Human and simulation results for Bock and Cutting (1992). Vertical bars represent the size of the attraction effect: the difference between the subject-attractor number match condition (the lower, circular endpoints) and mismatch condition (the higher, square endpoints). Error bars represent standard errors across the five randomly initialized models trained for each model architecture and training set. If the models simulate the relevant result from Bock and Cutting (1992), the attraction effect in RCs (the length of the solid red bar) is smaller than that in PPs (the length of the dashed blue-green bar). This pattern is reversed in LM-Only models trained on the WSJ Corpus, and no significant difference is found between modifier types in all other models.

A comparison of the human results and simulations using LM-Only and LM+CCG models is shown in Figure 5. Both types of models exhibited a significant attraction effect (LM-Only: *β* = 0.91, |*z*| = 34.19, *p* < 0.001; LM+CCG: *β* = 0.78, |*z*| = 24.14, *p* < 0.001). However, unlike humans, LM-Only models exhibited no interaction between the attraction effect and the type of modifier the attractor appeared in (*β* = −0.017, |*z*| = 0.66, *p* = 0.51). The LM+CCG models likewise showed no significant interaction (*β* = −0.058, |*z*| = −1.18, *p* = 0.07). The three-way interaction between attraction, syntactic environment (PP vs. RC), and model type (LM-Only vs. LM+CCG) found no evidence for any difference in the performance of the two types of models (*β* = 0.041, |*z*| = 1.00, *p* < 0.31). In summary, neither type of model successfully simulated the human pattern.

#### Simulation Results—Number Asymmetry.

Simulations using both models replicated the number asymmetry (LM-Only: *β* = 0.20, |*z*| = 5.47, *p* < 0.001; LM+CCG: *β* = 0.34, |*z*| = 7.40, *p* < 0.001). There was a significant 3-way interaction between attraction, subject number, and model type (*β* = −0.16, |*z*| = 2.66, *p* < 0.01), with LM+CCG exhibiting greater number asymmetry than LM-Only. In contrast to the effect of modifier type, then, the number asymmetry effect was captured by both types of models and was stronger in LM+CCG models.

#### Sensitivity to Training Corpus.

LM-Only models trained on the smaller WSJ Corpus displayed a significant attraction effect (*β* = 0.85, *p* < 0.001, |*z*| = 24.14), and an interaction between the attraction effect and the type of modifier (*β* = −0.09, *p* < 0.01, |*z*| = 2.63), such that attractors led to more errors when they were in relatives clauses than when they were in prepositional phrases. This effect was, crucially, in the opposite direction of that found in humans. Models trained on the larger Wikipedia dataset also exhibited an attraction effect (*β* = 0.94, *p* < 0.001, |*z*| = 8.32) but no interaction between that effect and modifier type (*β* = 0.0084, *p* = 0.76, |*z*| = 0.31). The Wikipedia-trained models exhibited a number asymmetry (*β* = 0.22, *p* < 0.001, |*z*| = 5.60), while WSJ Corpus-trained models did not (*β* = 0.053, |*z*| = 1.08, *p* = 0.28). The two types of models differed in the magnitude of the interaction between attraction and type of modifier, as assessed by a three-way interaction (*β* = 0.15, |*z*| = 2.29, *p* < 0.05); this was also the case for the analogous three-way interaction between model type, attraction and number (*β* = 0.10, |*z*| = 2.31, *p* < 0.05).

This pattern of results suggests a strong influence of dataset on the ability to replicate the difference in error rates between attractors in PPs and RCs, even with no difference in model architecture or training objective. While models trained on the smaller WSJ Corpus produced the wrong verb more often when the attractor was in an RC, models trained on the larger Wikipedia dataset showed no difference in error rates between the two conditions. While neither matched human behavior—more errors when attractors appear in PPs compared to RCs—training on Wikipedia resulted in more human-like results than training on the WSJ Corpus.

#### Overall Agreement Error Rates.

Human error rates, even in the conditions in which error rates were highest, were less than 15%. By contrast, models routinely made agreement errors in more than 50% of trials when an attractor was present. Though this difference in magnitude indicates that the models we trained are particularly susceptible to attraction errors, we take this discrepancy to be largely orthogonal to the goals of our investigation. We are concerned primarily with (1) whether our simple models exhibit agreement attraction (which high rates of agreement errors make apparent), (2) whether the factors we investigate modulate error rates in the same way in humans and models, and (3) whether changes to the models’ training data or training objective lead to more human-like behavior. Since these motivating questions consider only how differences in error rates change across various conditions, we have no reason to believe that high overall error rates are problematic for our analyses.

It is possible, of course, that modifications to our modeling setup that would reduce the overall error rate could imbue models with inductive biases that also affect differences in error rates across conditions. For instance, the LM-Only language models we use are chosen in part due to the fact that they do not “build-in” sophisticated syntactic representations (compared to, for instance, architectures that explicitly parse; Dyer et al., 2016). Since sophisticated syntactic representations are key to identifying the subject and avoiding agreement errors, the high rate of errors is tied directly to our choice of an small (in both number of parameters and quantity of training data), simple, and unbiased model for this evaluation.

#### GPT-2.

To address the concern with the LSTMs’ high overall agreement error rates, we repeat our simulations with GPT-2, a stronger model based on the Transformer architecture. Overall, GPT-2 error rates were smaller than, or roughly comparable to, human error rates in all conditions (ranging between 1.2% and 7.7%). GPT-2 exhibited agreement attraction (*β* = 0.23; |*z*| = 3.15; *p* < 0.005) as well as a number asymmetry (*β* = 0.24; |*z*| = 2.34; *p* < 0.05), but showed no interaction between the attraction effect and the type of modifier the attractor appeared in (*β* = 0.043; |*z*| = 0.59; *p* = 0.56). Thus, while GPT-2’s super-human overall error rates suggest that more powerful models can compute agreement more accurately overall, this increased overall accuracy does not necessarily lead to more human-like error patterns.

### Syntactic vs. Linear Distance Effects on Attraction

#### Background.

Franck et al. (2002) sought to further elucidate the role of syntactic structure in agreement attraction, focusing on a specific question: do the processes underlying agreement attraction operate over linear or hierarchical representations? To do so, they examined how attraction errors are affected by the linear distance between the attractor and verb, and compared the linear distance effect to the effect of the syntactic distance between those two words. Consider Example 11:

(11)  The threat(s) [_*PP*_ to the president(s) [_*PP*_ of the company(s)]] …

**Figure 6.**
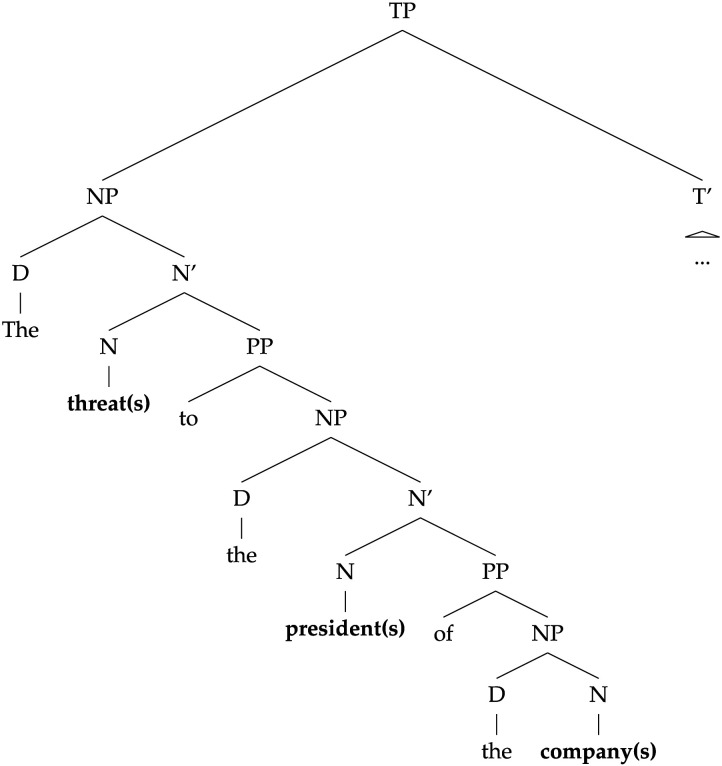
A simplified syntactic representation of Example 11. Even though the first attractor, the **president(s)**, is more distant from the eventual position of the verb (within the T′) than the second attractor, the **company(s)**, it is closer to the verb in the syntactic structure: fewer nodes need to be crossed to reach T′ from **president(s)**.

This sentence contains two potential attractors: the later one, *company(s)*, appears within a PP that modifies the earlier one, *president(s)*. Since the PP that contains *company(s)* is embedded within the PP that contains *president(s)*, the path from *company(s)* to the verb along the hierarchical structure of the sentence is longer than the path from *president(s)* to that verb (see Figure 6). If we find that the lengths of these paths—what Franck et al. (2002) call the *syntactic distance* between the attractor and the verb—are inversely proportional to the strength of the attraction effect caused by the two noun phrases, then we have evidence that attraction errors arise when participants process the hierarchical representations of the sentence. Franck et al. (2002) contrast these syntactic distances with the *linear distances* from the attractors to the verb. In terms of linear distance, *company(s)* is closer to the verb than *president(s)*, simply because *company(s)* appears to the right of *president(s)* in the linear sequence of words. Thus, by comparing the strength of attraction from the first, syntactically closer noun phrase (i.e., *president(s)*) to attraction from the second, linearly closer noun phrase (i.e., *company(s)*), we can investigate the nature of the structure (hierarchical or linear) used by humans or model during the agreement computations relevant to attraction: If the syntactically closer noun phrase causes stronger attraction than the linearly closer one, we have evidence for the role of hierarchical structure; if the difference is in the opposite direction, we have evidence for the role of linear order.

#### Human Results.

In Franck et al.’s (2002) experiment, syntactically closer attractors generated stronger attraction effects than linearly closer ones.

#### LSTM Simulations.

**Figure 7.**
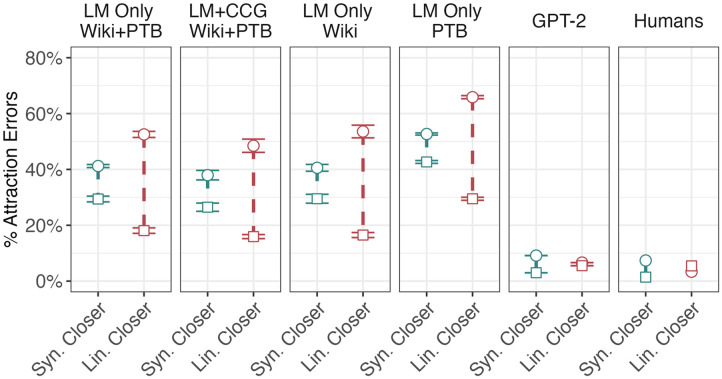
Human and simulation results for Franck et al. (2002). Vertical bars represent the size of the attraction effect: the difference between the subject-attractor number match condition (the lower, square endpoints) and mismatch condition (the higher, circular endpoints). These attraction effects are shown for the syntactically closer attractor (to the left of each facet) and the linearly closer attractor (to the right of each facet), marginalizing over the condition of the other attractor. Error bars for the LSTMs represent standard errors across the five randomly initialized models trained for each model training objective and training set. Crucially, in humans, the attraction effect from syntactically closer attractors is greater than that of linearly closer attractors. The reverse is true for all of the models with the exception of GPT-2.

The comparison of interest for each model is between the attraction effects caused by the syntactically closer attractor and that caused by the linearly closer attractor. Consequently, in Figure 7 we plot the magnitude of the attraction effect for each attractor, collapsing over the influence of the other attractor.

Both models displayed the opposite effect from humans: while there were significant effects of both the linearly closer attractor (LM-Only: *β* = 0.79, |*z*| = 38.51, *p* < 0.001; LM+CCG: *β* = 0.75, |*z*| = 33.57, *p* < 0.001) and the syntactically closer one (LM-Only: *β* = 0.29, |*z*| = 14.48, *p* < 0.001; LM+CCG: *β* = 0.28, |*z*| = 13.04, *p* < 0.001), linear effects were significantly stronger than syntactic ones (LM-Only: *χ*^2^ = 336.21, *p* < 0.001; LM+CCG: *χ*^2^ = 254.47, *p* < 0.001). A comparison between LM-Only and LM+CCG models did not find a significant difference in either the linearly closer or syntactically closer attractor’s attraction effect between model types (linearly closer: *β* = −0.020, |*z*| = 0.24, *p* = 0.80; syntactically closer: *β* = 0.013, |*z*| = 0.18, *p* = 0.86), again indicating that, contrary to our hypothesis, adding the CCG training objective did not make the models’ syntactic error patterns more human-like.

#### Effect of Training Corpus.

Both sets of models trained on only a single corpus showed a significant effect of attraction from both the syntactically closer attractor (WSJ: *β* = 0.20, |*z*| = 8.022, *p* < 0.001; Wiki: *β* = 0.26, *p* < 0.001, |*z*| = 12.65) and the linearly closer one (WSJ: *β* = 0.73, *p* < 0.001, |*z*| = −27.17; Wiki: *β* = 0.85, *p* < 0.001, |*z*| = 40.06). However, in both cases, as in our prior experiments, the attraction effect from linearly closer attractors was much stronger than the effect from syntactically closer attractors, the reverse of what Franck et al. (2002) found in humans (WSJ: *χ*^2^ = 205.82, *p* < 0.001; Wiki: *χ*^2^ = 442.64, *p* < 0.001). A comparison between the two models using two-way interactions revealed no significant differences in the attraction effect caused by either of the attractors (linearly closer: *β* = 0.050, |*z*| = 0.53, *p* = 0.595; syntactically closer: *β* = −0.021, |*z*| = 0.226, *p* = 0.82).

#### GPT-2.

GPT-2 showed a significant effect of attraction from both the syntactically closer attractor (*β* = 0.41; |*z*| = 8.88; *p* < 0.001) and the linearly closer attractor (*β* = 0.10; |*z*| = 2.42; *p* < 0.05). Unlike the other models we evaluated, GPT-2 did show stronger effects from the syntactically closer attractors (*χ*^2^ = 24.14; *p* < 0.001), as well as error rates across conditions (ranging from 1.92% to 9.20%) on par with those observed in Franck et al. (2002) (approximately 1.30–9.6%). In this case, then, GPT-2 was significantly closer to human behavior than our weaker LSTM-based models, suggesting that one of the differences between the models’ architecture of their training data aided in capturing syntactic distance effects.

### Linear Distance Effects in Disjunction

#### Background.

The two human experiments we have discussed so far suggested that agreement attraction in humans is sensitive to hierarchical syntactic structure, but neither provided clear-cut evidence as to whether or not humans are also sensitive to linear distance. In particular, in the Franck et al. (2002) comparison between linear and syntactic distance effects, syntactic distance was never held constant across linear distance conditions; as such, their results can speak only to the *relative* strengths of syntactic and linear distance, not to the existence of a linear distance effect independent of variation in syntactic distance. The absence of any linear distance effects in humans would indicate that agreement attraction errors—and, it follows, agreement computations—occur in the context of processes that operate over hierarchical structures, while the existence of a purely linear effect, over and above the hierarchical effects, would point to agreement being computed over a representation that encodes linear ordering.

To determine if there are such purely linear effects on agreement, Haskell and MacDonald (2005) compared rates of plural agreement in sentences where the subject was a disjunction (i.e. included the word *or*), and where one disjunct was singular and the other plural (see Examples 12 and 13). Both disjuncts are equally distant from the verb in syntactic terms^4^ but the second disjunct is linearly closer to the verb. As such, disjunction makes it possible to test for a linear distance effect independently of syntactic distance. Note that there is no canonical agreement pattern for disjunct subjects in Mainstream American English (see, for example, evidence from Foppolo & Staub, 2020), and thus neither the singular or plural form can be considered an agreement *error*.

(12)  Can you ask Brenda if the boy or the girls …
(13)  Can you ask Brenda if the boys or the girl …

#### Human Results.

Haskell and MacDonald (2005) found greater rates of plural agreement when the plural disjunct was linearly closer to the verb, indicating that linear distance affects agreement (though see Keung & Staub, 2018 for an alternative account of these results).

#### LSTM Simulations.

**Figure 8.**
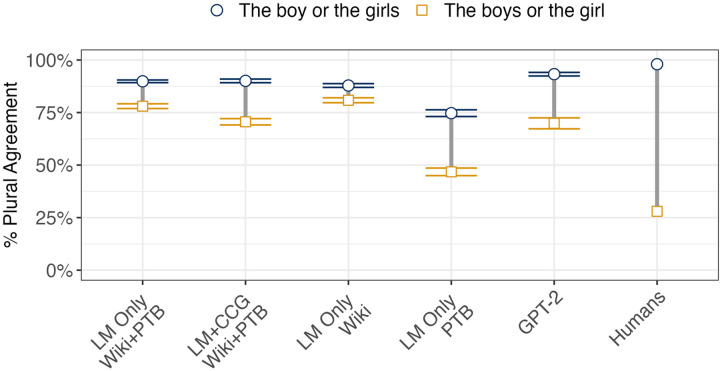
Human and simulation results for Haskell and MacDonald (2005). Vertical bars represent the size of the linear distance effect: the difference between plural agreement rates when the singular subject is closer to the verb position (the square endpoints) and when the plural subject is closer to the verb position (the circular endpoints). Error bars represent standard errors across the five randomly initialized models trained for each model architecture and training set. The size of the linear distance effect is represented by the length of the bar (all models had higher rates of plural agreement noun closer to the verb was plural than when it was singular). While all of the models exhibited some linear distance effect, the magnitude of the effect in humans was much larger than in any of the models.

Simulation results are shown in Figure 8. Both models exhibited a similar pattern to humans: conditions where the noun closer to the verb was plural had significantly greater rates of plural agreement than conditions where the noun closer to the verb was singular (LM-Only: *β* = −0.43, |*z*| = 11.22, *p* < 0.001; LM+CCG: *β* = −0.58, |*z*| = 12.84, *p* < 0.001). However, the size of the effect was much smaller than that reported in Haskell and MacDonald (2005), and thus this set of results, while promising, leaves room for other models to better match human behavior. A comparison across models indicated that the CCG supertagging objective strengthened the linear distance effect compared to LM-Only (*β* = 0.23, |*z*| = 4.03, *p* < 0.001). In this case, then, the syntactic objective did lead to more human-like behavior; surprisingly, this was the case for a linear distance effect rather than for a hierarchical one as we might have expected. We return to this point in the discussion.

#### Effect of Training Corpus.

Models trained on both smaller training sets also preferred to produce plural verbs when the plural disjunct appeared closer to the verb (WSJ: *β* = −0.64, |*z*| = 14.10, *p* < 0.001; Wiki: *β* = −0.23, |*z*| = 4.98, *p* < 0.001). The effect size was larger in models trained on the WSJ Corpus than in models trained on the much larger Wikipedia corpus (*β* = 0.46, |*z*| = 7.59, *p* < 0.001). This illustrates that training over larger datasets does not universally lead to more human-like behavior.

#### GPT-2.

Like all of the other models, GPT-2 preferred producing plural verbs when the plural disjunct was closer to the verb (*β* = −0.75; |*z*| = 8.69; *p* < 0.001). The magnitude of this effect in GPT-2 was comparable to that found in some of the more human-like LSTM-based models (LM+CCG and LM-Only models trained on WSJ), but was still far below that observed in humans. Since there is no canonical grammatical response in this experiment, we cannot determine whether GPT-2’s sophisticated architecture led to a reduction in error rates in this simulation.

### Notional Number and Distributivity

#### Background.

The previous experiments have characterized syntactic effects on agreement attraction: How does the linear and hierarchical position of the attractor influence agreement behavior? We now turn to semantic factors that affect agreement processing. Several studies have demonstrated an influence of *semantic* or *notional number*—the number of countable parts in the conceptual entity referred to by the noun phrase. Notional number contrasts with *grammatical number*, which is typically determined by the morphology of the head noun (e.g., the plural morpheme *-s* in many varieties of English). The role of notional number is particularly salient in collective NPs:

(14)  The gang near the motorcycles …
(15)  The gang on the motorcycles …

In Example 14, the preposition *near* tends to give rise to a *collective* reading, where the gang is viewed as a single collective entity located near a group of motorcycles. This gives the NP a singular notional number. By contrast, the preposition *on* in Example 15 favors a *distributive* reading, where each member of the gang is located on their own motorcycle; this results in plural notional number.

While subject-verb agreement is ostensibly a syntactic constraint, prior work has demonstrated that it is also affected by the notional number of the subject, with notionally plural subjects leading to higher rates of plural agreement than notionally singular subjects (Bock et al., 1999; Eberhard, 1999; Humphreys & Bock, 2005). Analyzing the ability of neural language models to simulate these notional number effects is of particular interest given that the models are trained solely on word prediction or CCG supertagging; since models only understand language through the text they are trained on, they lack the grounding in the physical world that might be necessary to capture agreement patterns that depend on factors such as the spatial organization of gang members and motorcycles (Bender & Koller, 2020). Given such impoverished semantic capabilities, we hypothesize that the models will have greater difficulty capturing these semantic influences on human agreement behavior.

#### Human Results.

In a sentence completion study, Humphreys and Bock (2005) found that participants produced plural verbs more often when the preposition favored a distributive reading (as in Example 15) than when it favored a collective reading (as in Example 14).

#### LSTM Simulation Results.

**Figure 9.**
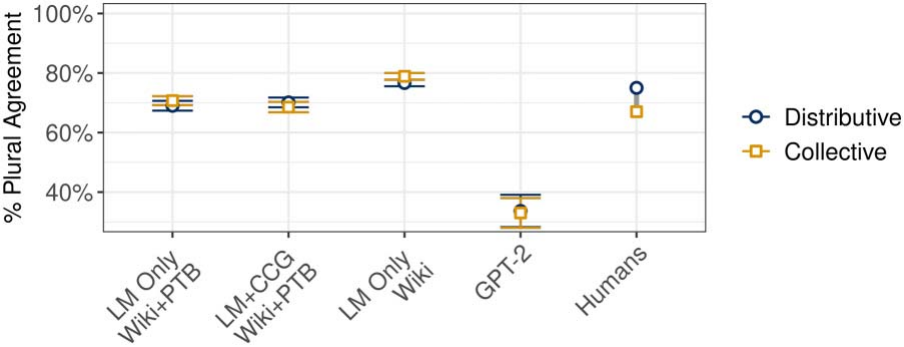
Human and simulation results for Humphreys and Bock (2005). Endpoints represent the rate of plural agreement in the distributive-biased condition (circular endpoints) or the collective-biased condition (square endpoints). Error bars represent standard errors across the five randomly initialized models trained for each model architecture and training set. In humans, Humphreys and Bock (2005) observed higher rates of plural agreement when the reading of the collective subject was biased toward a distributive reading. We observe no such difference in any of the models’ results.

We compare plural agreement rates for humans and both types of LSTMs in Figure 9. Models showed no significant difference in rates of plural agreement between distributive-biased and collective-biased prepositions (LM-Only: *β* = 0.047, |*z*| = 1.32, *p* = 0.19; LM+CCG: *β* = −0.030, |*z*| = 0.65, *p* = 0.52), and there was no evidence of an interaction that would indicate a difference between the two types of models (*β* = 0.074, |*z*| = 1.29, *p* = 0.20). These null results could indicate one of two things: either our models do not use representations of notional number as part of the computations that result in an inflected verb form, or they simply have no representation of notional number at all. We will examine the second possibility in the Summary of Results.

#### GPT-2.

Like in our simulation of linear distance effects with disjunct subjects, there is no canonical grammatical response we should expect our models to have, so we cannot test whether the model’s correctness improves. Like the other models, GPT-2 showed no differences in the rates of plural agreement between the two types of prepositions (*β* = −0.017; |*z*| = 0.21; *p* = 0.83).

### Argument Status

#### Background.

Agreement attraction is also affected by factors at the interface of syntax and semantics. Building on the hypothesis that *core arguments*, which are necessary for the interpretation of the verb, are encoded in memory more distinctively than *oblique arguments*, Parker and An (2018) hypothesized that the strength of attraction would differ between attractors in core arguments and attractors in oblique arguments:

(16)  Core argument: The waitress who sat **the girl(s)** unsurprisingly was/were unhappy about all the noise.
(17)  Oblique argument: The waitress who sat **near the girl(s)** unsurprisingly was/were unhappy about all the noise.

The reasoning that underlies this prediction is as follows. Memory retrieval models argue that agreement errors are caused by erroneous retrieval of the attractor’s number feature instead of that of the subject (Badecker & Kuminiak, 2007; Parker & An, 2018; Wagers et al., 2009). These misretrieval errors are less likely if the features of the attractor are well encoded, which, by hypothesis, they are in core arguments but less so in oblique ones (Parker & An, 2018; Van Dyke & McElree, 2011): More strongly encoded features provide a stronger indication that the attractor is not the subject, steering the memory retrieval process away from the attractor.

Parker and An (2018) presented participants with sentences such as Example 16 and 17 in a self-paced reading paradigm. The study followed a 2 × 2 × 2 design: singular vs. plural attractor, grammatical vs. ungrammatical sentence (i.e., singular vs. plural main verb; the subject was always singular), and core vs. oblique argument.

Recall that in self-paced reading, agreement attraction can manifest in two ways: first, as a facilitatory effect in ungrammatical sentences, where an ungrammatical sentence is read faster in the presence of an attractor NP that mismatches the subject in number (and thus matches the verb in number). The attractor creates an illusory agreement dependency with the verb, which shares a number feature with it. Thus, in the case of an attraction error, an ungrammatical sentence is read as if it were a grammatical one, leading to shorter reading times than if no error had occurred. Second, agreement attraction can manifest as an inhibitory effect in grammatical sentences, where grammatical sentences are read more slowly in the presence of an attractor NP whose number mismatches the subject (and therefore also the verb). An agreement error in these circumstances would result in an ungrammatical agreement relation, as the attractor and verb do not share the same number, which in turn would result in longer reading times than if no error had occurred. Overall, the attractor’s presence reduces the processing cost associated with ungrammaticality—the difference between reading times in grammatical and ungrammatical conditions. In the Parker and An (2018) paradigm, we expect this reduction in the cost of ungrammaticality to surface at the matrix verb (*was*/*were*), where the grammaticality of the agreement dependency can be determined.

#### Human Results.

In Parker and An’s (2018) experiment, participants were more susceptible to attraction errors in ungrammatical sentences when the attractors were in oblique arguments than when they were in core arguments. Parker and An (2018) do not report an analysis of reading patterns on grammatical sentences.

#### LSTM Simulation Results—Ungrammatical Sentences.

**Figure 10.**
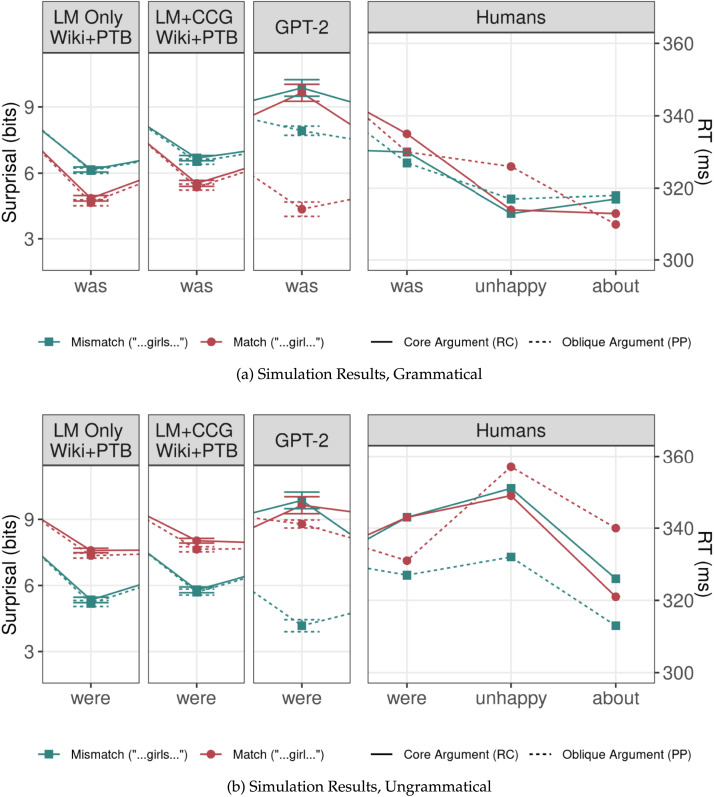
Word-by-word surprisals from our simulations and corresponding reading times from Exp. 1 of Parker and An (2018). Error bars are standard errors. Since effects in self-paced reading typically spill over into the reading times of the next few words, we provide two additional words for the human results. The relevant effect is found at *unhappy* in the human data, with the attraction effect in the oblique argument condition (the difference between dashed lines) being significantly larger than the attraction effect in the core argument condition (the difference between solid lines). We see no such difference in models other than GPT-2.

A comparison of surprisals at the critical word to the mean reading times reported by Parker and An (2018) can be found in Figure 10; for full word-by-word surprisals, and in particular the differences in surprisal at the attractor, see Appendix D. As in the human experiment, both models showed an attraction effect for ungrammatical oblique argument sentences (LM-Only: *β* = −1.09, |*t*| = 26.11, *p* < 0.001; LM+CCG: *β* = −0.97, |*t*| = 19.17, *p* < 0.001). Unlike humans, however, the models also showed attraction effects for ungrammatical core argument sentences (LM-Only: *β* = −1.12, |*t*| = 27.80, *p* < 0.001; LM+CCG: *β* = −1.12, |*t*| = 22.19, *p* < 0.001), and there was no significant interaction between argument status and attraction (LM-Only: *β* = −0.018, |*t*| = 0.615, *p* = 0.53; LM+CCG: *β* = −0.072, |*t*| = 1.94, *p* = 0.051). An analysis comparing LM-Only and LM+CCG models did not find a significant three-way interaction between model type, argument type and number mismatch (*β* = 0.053, |*t*| = 1.12, *p* = 0.26), suggesting that the syntactic training objective did not affect the models’ ability to simulate the human error patterns.

#### LSTM Simulation Results—Grammatical Sentences.

As Parker and An (2018) do not present attraction analyses for the grammatical sentences in their experiment, we present the simulation results here without comparing them to the human patterns. Both models showed a significant effect of attraction (LM-Only: *β* = 0.69, |*t*| = 24.00, *p* < 0.001; LM+CCG: *β* = 0.57, |*t*| = 15.62, *p* < 0.001), but no significant interaction between attraction and argument status (LM-Only: *β* = −0.037, |*t*| = 1.28, *p* = 0.20; LM+CCG: *β* = −0.0024, |*t*| = 0.064, *p* = 0.95). A comparison between LM-Only and LM+CCG did not find a three-way interaction between the additional objective, attractor argument type, and subject-attractor number match (*β* = −0.034, |*t*| = 0.73, *p* = 0.46). It did, however, yield an interaction between the model type and subject-attractor number match, reflecting smaller attraction effects in LM+CCG (*β* = −0.0012, |*t*| = 2.15, *p* < 0.05).

#### GPT-2.

For this (and the following) simulation of a comprehension experiment, there is no real measure of a model’s error rate. As a result, these results cannot show whether GPT-2 has a lower overall error rate relative to our LSTM models. We thus present results of these simulations only to evaluate the ability of GPT-2 to mimic human error patterns.

In ungrammatical sentences, we found a significant attraction effect (*β* = −1.10; |*t*| = 7.01; *p* < 0.001), with an interaction with argument status such that the attraction effect was attenuated when the attractor was in core arguments compared to oblique arguments (*β* = 1.21; |*t*| = 7.71; *p* < 0.001). Grammatical sentences displayed a similar pattern, with a significant attraction effect (*β* = 0.94; |*t*| = 5.70; *p* < −0.001) that was smaller when the attractor was in a core argument (*β* = −0.83; |*t*| = 5.039; *p* < 0.001). Unlike the other models, and like human participants, GPT-2 showed an effect of argument status on the strength of attraction. This suggests that some aspect of GPT-2’s training or architecture may allow GPT-2 to represent argument status and encode that feature in a way that influences agreement processing.

### Grammaticality Asymmetry

#### Background.

As noted in the previous section, attraction can affect reading in two ways: it can cause participants to read grammatical sentences more slowly, or it can cause them to read ungrammatical sentences faster. Theories that attribute agreement attraction to an error in encoding the number of the subject (Eberhard et al., 2005, among others) predict that both of these effects should be of the same magnitude (Badecker & Kuminiak, 2007; Wagers et al., 2009). This is because grammaticality is determined by the number of the verb, which appears only after the subject is encoded; as such, there is no reason to expect subject encoding errors to occur with different frequency in grammatical and ungrammatical sentences.

Some encoding accounts also hypothesize that encoding errors emerge from an erroneous percolation of the attractor’s number feature to the subject noun phrase as a whole (Franck et al., 2002). These accounts thus additionally predict that attraction errors can only occur when the attractor is within the subject NP, as that is the only case in which there is an upward path through which the attractor’s number feature can percolate to the subject node.

Wagers et al.’s (2009) self-paced reading study tests both of these predictions using sentences with RC-modified subjects:

(18)  The musician(s) [ who the reviewer(s) praise(s) so highly ] will probably win a Grammy.

Unlike the sentences used in the Bock and Cutting (1992) experiment discussed above, in these materials it is the matrix clause subject, *musician(s)*, that acts as the attractor NP, and the agreement relation that is manipulated—the subject-verb dependency between *reviewer(s)* and *praise(s)*—is internal to the relative clause. As a result of this configuration, the attractor is not within the subject, and thus percolation accounts predict no attraction in this paradigm.

#### Human Results.

Contrary to the predictions of all encoding accounts of agreement attraction, Wagers et al. (2009) found that human readers show a *grammaticality asymmetry*: they displayed attraction effects in ungrammatical sentences, but not in grammatical ones. Wagers et al. (2009) additionally confirmed that attractors outside of a relative clause can cause attraction within that relative clause, providing additional evidence against the percolation-based encoding account in particular.

#### LSTM Simulation Results.

**Figure 11.**
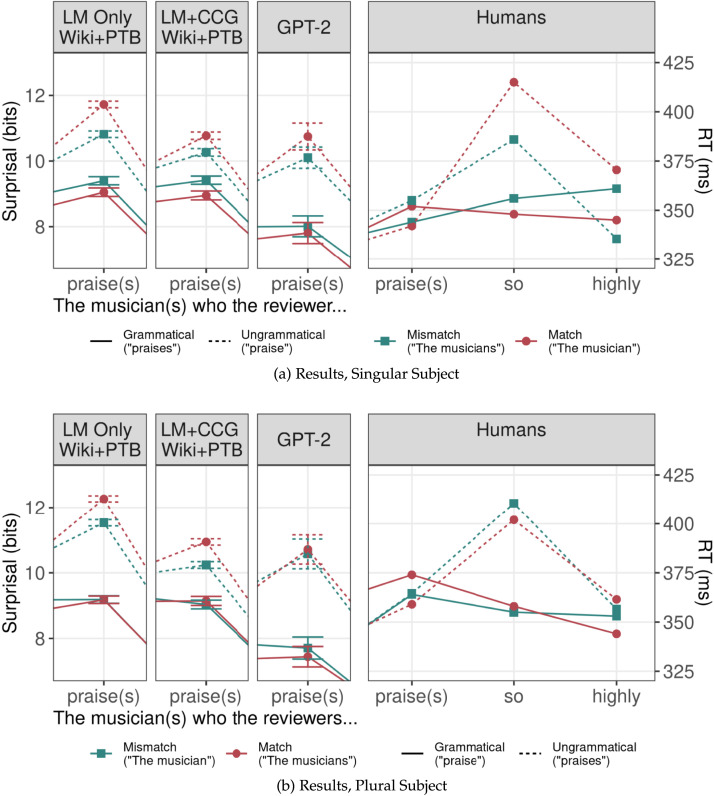
Surprisals for models in our simulation of Exp. 3 of Wagers et al. (2009) at the verb *praise(s)*, where the grammaticality of the agreement relation within the RC becomes clear, compared to the human data from that experiment (right). Error bars are standard errors. We see a grammaticality asymmetry in both humans and models, reflected in that fact that attraction in ungrammatical sentences (the difference between the dashed lines) is stronger than in grammatical sentences (the difference between the solid lines).

A comparison between the models’ surprisals at the critical word and reading times at the critical region of the human data can be seen in Figure 11. For full word-by-word surprisals, including surprisal differences due to words prior to the critical region, see Appendix D. Like humans, both types of models showed a significant agreement attraction effect in ungrammatical sentences (LM-Only: *β* = −0.41, |*t*| = 12.48, *p* < 0.001; LM+CCG: *β* = −0.30, |*t*| = 10.17, *p* < 0.001), but, unlike humans, they also showed attraction in grammatical sentences (LM-Only: *β* = 0.09, |*t*| = 3.32, *p* < 0.005; LM+CCG: *β* = 0.089, |*t*| = 3.02, *p* < 0.005). We found a significant interaction between attraction and grammaticality in both models (LM-Only: *β* = −0.16, |*t*| = 6.72, *p* < 0.001; LM+CCG: *β* = 0.107, |*t*| = 4.83, *p* < 0.001), such that ungrammatical sentences displayed larger attraction effects than grammatical ones, in line with the grammaticality asymmetry observed in humans. An analysis comparing the simulation results across types of models found no evidence of an effect of the CCG supertagging objective on the grammaticality asymmetry (*β* = −0.054, |*t*| = 1.57, *p* = 0.11). The presence of an asymmetry indicates that, like in humans, agreement errors in models are not simply caused by faulty encoding of the subject’s number, but by a mechanism that is sensitive to the verb’s number. This could take the form of a retrieval error, as Wagers et al. (2009) argue is the case for humans, or a bias toward reading sentences as grammatical (Hammerly et al., 2019). We return to this point in the summary of results.

#### GPT-2.

Unlike the rest of the models we evaluated, GPT-2 failed to display a significant attraction effect in either ungrammatical sentences (*β* = 0.39; |*t*| = 1.46; *p* = 0.15) or grammatical sentences (*β* = −0.23; |*t*| = 1.18; *p* = 0.24), and there was no significant interaction between attraction and grammaticality (*β* = −0.16; |*t*| = 0.44; *p* = 0.66). In this case, then, the weaker LSTM models were more human-like than the stronger transformer model GPT-2. We did find a significant attraction effect in the subset of sentences with a singular subject, and thus a plural attractor in the mismatch condition (*β* = 0.65; |*t*| = 2.33; *p* < 0.05); this is the condition where we would expect the largest attraction effects due to a combination of number asymmetry and grammaticality asymmetry (this analysis replicates one of the simulations reported by Ryu & Lewis, 2021).

### Summary of Results

The simulations we reported in this section aimed to answer three major questions: first, what phenomena from the human agreement attraction literature are captured by a simple neural network language model without explicit syntactic supervision or syntactic inductive bias (LM-Only)? Second, does the addition of the explicit syntactic training objective lead models to better capture those phenomena? And third, how do differences in the corpora used to train a neural language model affect the agreement attraction phenomena the model captures? In this section, we discuss how the results of our six simulations bear on these three questions. We then contextualize our findings more broadly in the General Discussion.

#### What Phenomena Do LM-Only Models Capture?

Our first goal was to determine how well a simple language model that lacks explicit language-specific biases captures the range of factors that affect agreement processing in humans. To do so, we compared the behavior of human participants to the behavior of LM-Only models trained on the combination of Wikipedia and the WSJ Corpus. The experiments we simulated can be grouped into three categories: experiments that bear on the role of hierarchical structure in agreement processing, experiments that bear on the role of semantic factors in agreement processing, and an experiment that demonstrates a grammaticality asymmetry in agreement attraction. We will discuss the effect of additional syntactic training in the next section.

##### The Grammaticality Asymmetry.

In our simulation of Experiment 3 from Wagers et al. (2009), we sought to determine whether models can simulate the grammaticality asymmetry, where attractors cause ungrammatical sentences to be read faster but do not cause grammatical sentences to be read more slowly. We found that models—both LM-Only and LM+CCG—behave in line with this asymmetry, displaying greater susceptibility to attraction in ungrammatical than grammatical sentences.

Wagers et al. (2009) interpret the grammaticality asymmetry in humans as indicating that attraction does not result solely from encoding errors. In English, subjects generally precede the verbs they agree with. As a result, an error in encoding the subject’s number necessarily occurs before the verb is processed, and therefore the number of the verb—which determines the grammaticality of the subject-verb agreement relation—should not affect the rate of agreement errors: we should see as many errors in grammatical sentences as in ungrammatical ones. The fact that we do see a grammaticality asymmetry, Wagers et al. (2009) argue, supports models that attribute agreement attraction to erroneous retrieval of the subject’s number at the verb rather than erroneous encoding of the subject.

Wagers and colleagues’ account of the grammaticality asymmetry could plausibly explain our LSTM models’ behavior. These models can be divided into two components: an LSTM encoder, which constructs a representation of the sequence of words observed thus far, and a decoder, which takes the representation generated by the encoder and outputs a probability distribution over the next word. The distinction between these two components roughly corresponds to the distinction between encoding and retrieval processes: when constructing its encoding, the LSTM encoder only has access to the subject, as is the case for encoding processes in human participants. By contrast, the decoder’s estimate of a verb’s likelihood as the next word depends on the identity of the verb: our models’ estimate of *P*($w_{i+1}^{*}$ | *w*_1_, …, *w*_*i*_) is sensitive to the hypothetical next word $w_{i+1}^{*}$. Since this probability is directly mapped to our simulated behavioral measure (as described in the Methods section), we can use Wagers and colleagues’ reasoning to conclude that some of the erroneous behavior of the models must be attributed to the decoder rather than the encoder: the asymmetry can only arise if the process generating the errors can determine the number (and thus the grammaticality) of the verb.

##### Factors at the Syntax-Semantics Interface.

We simulated two human experiments that were concerned with factors at the syntax-semantics interface: distributivity in agreement with collective subjects (Humphreys & Bock, 2005) and the effect of argument structure on agreement attraction (Parker & An, 2018). Both LSTM models failed to mirror human behavior: there was no difference in plural agreement rates between distributive-biased and collective-biased subjects, and no difference in attraction rates between attractors in core and oblique arguments. We hypothesize that models’ failure to simulate these semantic effects on agreement is connected to a more fundamental issue in language models: the inability of models trained solely on language modeling to develop the grounding necessary for true language understanding (Bender & Koller, 2020). In particular, to match the hypothesized mechanism underlying human behavior for the distributivity experiments (Humphreys & Bock, 2005), a model would need to distinguish between, for example, an NP that is more likely to be conceptualized as a single, collective entity and an NP that is more likely to be conceptualized as multiple entities distributed in space. This kind of mapping, from linguistic material to entities in an external world, may lie beyond the abilities of models trained solely on linguistic material at this scale (though see Pavlick, 2023 for evidence that these capacities may emerge when models are trained on orders of magnitude more training data). We speculate that a multi-modal model with a visual training objective may be better able to capture such effects (for a example of a multi-modal model in distributional semantics, see Bruni et al., 2014).

Similar limitations may underlie the models’ failure to simulate the results of Parker and An (2018). The difference between attractors in core and oblique arguments in humans is hypothesized to be due to the differential encoding of arguments based on their importance during interpretation: since core arguments are more central to interpretation than oblique ones, attractors in core arguments are better encoded (Van Dyke & McElree, 2011), and thus are less likely to interfere with agreement than more poorly encoded oblique arguments. Since word prediction models are never explicitly tasked with interpreting the meaning of the representations they construct—only with predicting upcoming words—they are less subject to the pressures that Parker and An (2018) suggest lead humans to differentially encode core and oblique arguments. This may partly explain why this distinction does not affect the models’ agreement error rates. However, this explanation is complicated by our GPT-2 simulations, which did reveal differences in attraction from core and oblique arguments. We leave an exploration of exactly how this behavior manifests in GPT-2 to future work.

##### Hierarchical Structure and Linear Distance.

The first three experiments we simulated characterized the effect of syntactic and linear position on agreement attraction: differences in attraction strength between attractors in prepositional phrases and relative clauses (Bock & Cutting, 1992), differences in syntactic distance between the attractor and verb (Franck et al., 2002), and differences in the linear distance separating disjuncts in the subject from the verb (Haskell & MacDonald, 2005). LM-Only models broadly failed to capture these structural effects: they showed no difference in attraction strength between PP and RC attractors, whereas humans made more attraction errors for preambles with PP attractors compared to those with RC attractors (Bock & Cutting, 1992). Our simulations also showed stronger attraction effects from attractors linearly closer to the verb than ones that were syntactically closer to the verb—the reverse of the effect found by Franck et al. (2002). Taken together, these two results suggest that models operate over linear representations based on the surface form of the input rather than the hierarchical representations used by humans (Momma & Ferreira, 2019). Finally, though the models displayed a significant effect of linear distance in the same direction as the effect found by Haskell and MacDonald (2005), the magnitude of this effect was far smaller than in humans.

**Figure 12.**
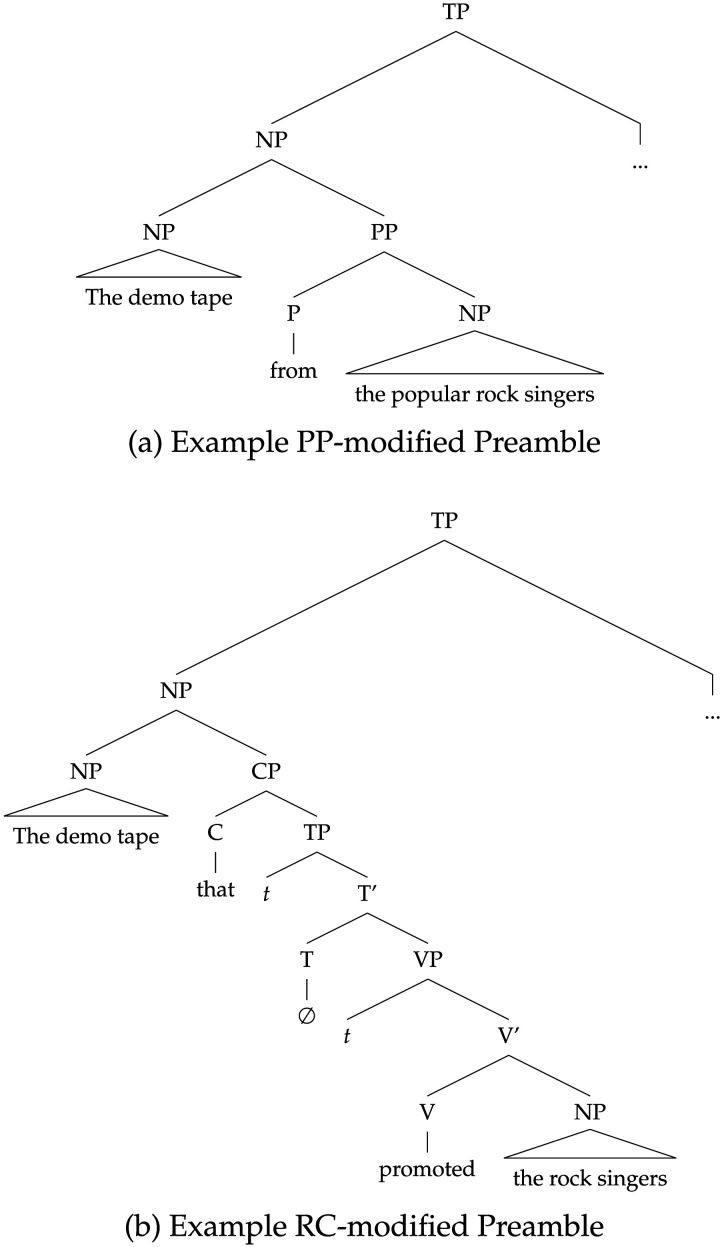
Example (simplified) syntactic trees corresponding to the PP and RC conditions in Bock and Cutting (1992). Crucially, the attractor NP in embedded more deeply in the subject’s structure in the RC-modifier condition (12b) than in the PP-modifier condition (12a), resulting in a longer syntactic distance from the attractor to the inflected verb’s position.

We hypothesize that stronger hierarchical biases may be necessary for models to fully simulate syntactic and linear distance effects on human agreement processing. The two empirical findings we failed to capture—the effect of the type of modifier in which the attractor appears (PP vs. RC), and the effect of the depth of the attractor within the subject—can both be explained through syntactic distance (Franck et al., 2002), under the assumption that higher rates of agreement errors correspond to a shorter distance from the attractor to the verb in the hierarchical structure of the sentence (see Figure 12). This suggests that what may be missing from our models is an accurate hierarchical representation of input that has a strong causal role in the models’ word predictions: if the models compute agreement over a flat, linear representation, they cannot be sensitive to differences in a measure such as syntactic distance. Our LM+CCG models, which were trained with explicit syntactic supervision, were motivated by this hypothesis; we discuss those models in the next section.

#### Does the Syntactic Bias Imparted by Supertagging Lead to More Human-Like Behavior?

Success at the supertagging task requires sophisticated representations of syntactic structure. For example, correctly predicting the supertag (S\NP)/ADJ for “is” in “The key to the cabinets is …” requires a model to both recognize an NP to its immediate left and predict that the upcoming material will eventually result in an ADJ that combines with the current word and the NP to the left to form an S. That is, the model must identify “the cabinets” or “the key to the cabinets” is an NP, predict that the next word is likely to be an ADJ like “rusty,” and reason that “is” must be an (S\NP)/ADJ to have the full sentence (“The key to the cabinet is rusty”) form an S. We hypothesized that a language model that shared the representations it uses for word prediction with a supertagger would be biased toward accessing the syntactic information in those representations, and, as a result, would exhibit more human-like error patterns when simulating agreement attraction experiments, particularly those that tested syntactic phenomena (Bock & Cutting, 1992; Franck et al., 2002). This hypothesis was not borne out: the syntactic training objective had no discernible impact on the ability of the models to capture human error patterns in our simulations of Bock and Cutting (1992) and Franck et al. (2002). At the same time, this objective did lead to more human-like results in simulation of experiments that investigated non-syntactic factors: LM+CCG models exhibited a stronger number asymmetry (Bock & Cutting, 1992), stronger linear distance effects (Haskell & MacDonald, 2005), and weaker attraction in grammatical sentences (Parker & An, 2018) than LM-Only models. We discuss each of these observations in turn.

##### Are Representations Shared Between Word Prediction and Supertagging?

Why did the supertagging objective fail to affect the networks’ syntactic behavior? Our hypothesis was that in the multi-task setting the representations generated by the LSTM encoder would better encode fine-grained syntactic information; those, in turn, would be used not only by the classifier that performed the supertagging task, but also by the classifier dedicated to word prediction, which determines the overall behavior of the cognitive model. This hypothesis crucially rests on the assumption that the representations used by the two classifiers are shared; if that assumption is incorrect, and the two sets of representations are distinct, separable subspaces of the LSTM encoder’s representational space, we would expect little difference in the syntactic behavior of LM-Only and LM+CCG models during word prediction.

**Figure 13.**
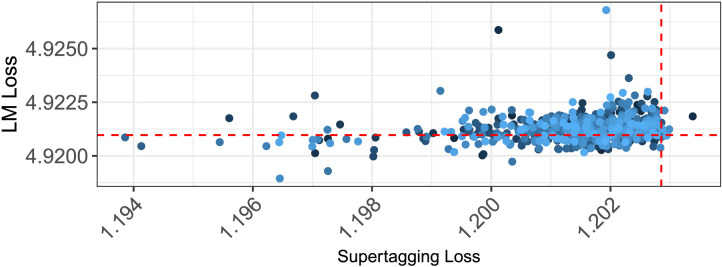
The language modeling and CCG supertagging losses over the test set of one of our LM+CCG models with the output of one neuron in the final layer set to 0. Each dot represents the performance of the model ablating a particular final-layer neuron. Dashed lines represent the model’s performance with no neurons ablated. Lower losses indicate better performance.

To test whether the limited impact of the supertagging objective was due to a lack of shared representations between the two objectives, we conducted two analyses: a local ablation analysis and a distributed “amnesic probing” analysis (Elazar et al., 2021). The local ablation analysis aimed to determine whether the outputs of particular neurons encoded properties that were crucial to performance in both word prediction and CCG supertagging. To do this, we measured the performance of one of our LM+CCG models over the test set of CCGBank after ablating (i.e., setting to 0) in turn each of the 650 neurons in the output layer of our model. This is equivalent to ignoring the information encoded in one of the dimensions of the models’ vector representation of the input. If the features encoded by one of these neurons is shared across the two tasks, removing the output of that neuron from the model’s representation should impact the performance of our model on both of those tasks. By contrast, removing the output of a neuron that encodes features that are used in just one of the models’ tasks should only affect the model’s performance on that task. We plot the results of this analysis in Figure 13. We find a positive correlation between word prediction and supertagging losses (*r* = 0.21; *t* = 5.44, *p* < 0.001), indicating that intervening on a neuron tends to affect word prediction and supertagging losses in the same way. This suggests that representations are largely shared between the language modeling and supertagging components of our models.

Interpreting this first analysis depends on a localist interpretation of the networks’ representations—it assumes that each individual neuron encodes some potentially syntactic information that we can remove and observe performance after that information has been removed. While this approach has been fruitful in isolating meaningful units of syntactic information in some cases (Lakretz et al., 2019, 2021), representations emerging from neural networks need not represent information in this highly localized manner (Rumelhart & McClelland, 1987).

To address the possibility that the relevant representations are distributed, we use amnesic probing (Elazar et al., 2021), an approach that uses techniques from the de-biasing literature in Natural Language Processing (Bolukbasi et al., 2016; Ravfogel et al., 2020) to identify and remove differences across a linear subspace of a models’ representational space, preventing the model from using particular sources of information.

In practice, our procedure takes the form of a single step of the Iterated Null Space Projection (INLP; Ravfogel et al., 2020) method using the trained CCG decoder as the classifier whose accuracy we wish to reduce: we construct a linear transformation *T* from our trained linear classifier *C* such that for any vector representation *x*, *C*(*T*(*x*)) = 0, and apply *T* to to all vector representations output by our model. Since the classifier trained to predict CCG supertags can no longer distinguish between vector representations transformed by *T*, we can conclude that all information formerly used to perform CCG supertagging was stripped from our model’s representations. If information is shared across the word prediction and supertagging tasks, then we should expect applying *T* to reduce word prediction performance.

Of course, for this and the previous analysis, it is necessarily the case that some information will be useful to both tasks: for example, removing a representation of the identity of the previous word will impair both word prediction and the identification of that previous word’s supertag. What we are interested in is how much information *learned from the CCG supertagging training* is used during language modeling. To set an upper bound on the reduction in performance that could be attributable to information the model learned to represent through just language modeling training, we trained a supertagging classifier over the representations from one of our LM-Only models. Crucially, only the final classifier was trained on CCG supertags: the LM-Only model generated a representation based only on its word prediction training, and a classifier (identical in architecture to the supertagging classifier in our LM+CCG models) was trained to predict supertags from those LM-Only representations. In other words, the weights of the LM-Only encoder were frozen before training the classifier, and thus the classifier could only use the representations learned from the word prediction objective. We then applied the amnesic probing procedure to this model, removing any information useful to CCG supertagging that was learned solely from word prediction. The drop in language modeling performance we observe after this procedure acts as a baseline of performance loss that is due to the removal of features that are *not* learned as part of supertagging training. The results of this analysis are shown in Table 1.

**Table 1.** Word prediction losses (lower is better) and CCG supertagging accuracy (higher is better), before and after amnesic probing techniques were used to remove CCG-related information from the models’ representations.

Model	LM Loss	CCG Accuracy
LM+CCG	4.921	84.5%
LM+CCG, amnesic	7.180	21.23%
LM-Only	4.325	84.30%
LM-Only, amnesic	7.182	21.23%

We observe two things from these results. First, amnesic probing affects LM-Only models as strongly as LM+CCG models, if not more strongly. This could suggest that the information learned from CCG supertagging training of LM+CCG models is not used during language modeling. However, we also see that the classifier trained over the representations generated by our LM-Only models achieves similar top-1 accuracy to our LM+CCG models. This suggests that the syntactic information in the encoder’s representations that is learned in the LM+CCG setting training is already learned through word prediction alone. This suggests that the failure of the CCG supertagging objective to lead to more human-like syntactic behavior may simply be due to the fact that the CCG supertagging task is insufficiently syntactically complex to improve our models’ syntactic representations beyond those learned from simple word prediction. We will discuss the potential implications of this hypothesis, as well as how more syntactically sophisticated tasks may overcome this issue, in the General Discussion.

##### When Do LM+CCG Models Better Simulate Humans Than LM-Only Models Do?

While we found little difference between LM-Only and LM+CCG models in the simulations that bear on linear and syntactic distance, we did find three notable differences between the models’ performance, all of which bring LM+CCG models closer to the human error patterns.

First, in our simulation of Bock and Cutting (1992), LM+CCG models exhibited a larger number asymmetry than LM-Only models (like humans, both models showed a larger attraction effect for plural attractors than for singular attractors). Second, in our simulation of Haskell and MacDonald (2005), LM-Only models, like humans, showed a bias in favor of agreeing with the number of the linearly closer attractor in a disjunct subject like *the boys and the girls*. However, the magnitude of this effect was much smaller than was observed that in human participants. LM+CCG models showed a larger effect size for this experiment, though it was still not comparable to that of humans. Finally, in our simulation of Parker and An (2018), LM+CCG models showed smaller agreement attraction effects in grammatical sentences than LM-Only models, while the attraction effect in ungrammatical sentences did not change significantly between LM-Only and LM+CCG models. The pattern shown by LM+CCG models is in line with the grammaticality asymmetry observed in the human experiments of Wagers et al. (2009), where agreement attraction was found only in ungrammatical sentences.

To understand these differences in light of our analysis of shared representations, it is helpful to consider the various ways in which an additional supertagging objective can influence our model’s word prediction behavior. We hypothesized that supertagging would give the model additional incentive to learn syntactic representations that will then be recruited for word prediction. Our analysis in the previous section suggests that this has not happened, since the LM+CCG models rely on the same syntactic information learned just by training on next-word prediction.

However, there are other, indirect ways in which this additional training task can influence the representations a model learns. For instance, additional pressure for performance on CCG supertagging may not lead to new information being encoded, but may reduce pressure to learn other information used only in language modeling. Since the models’ loss is a sum of language modeling and CCG supertagging losses, The optimization process will prefer robustly encoding information that helps both training objectives to encoding information that only marginally improves language modeling performance. This could result in weaker, more heuristic sentence processing capacities that lead to the more human-like error patterns we observe.

#### How Does Training Data Affect Agreement Behavior?

Next, we discuss our experiments that compared LM-Only models trained on the Wall Street Journal section of the Penn Treebank (WSJ) to those trained on a subset of English Wikipedia. These two training corpora differ in both size and genre, both of which could affect the agreement behavior our models exhibit; we will discuss these factors in turn.

The first difference between the corpora is size. Whereas the WSJ corpus is composed of just under 1 million words, the subset of English Wikipedia is significantly larger, consisting of approximately 80 million words. In general, models that are given more data learn to perform better at word prediction (Kaplan et al., 2020), and models that perform better at their task tend to behave in a more human-like manner (Goodkind & Bicknell, 2018; Merkx & Frank, 2021, though see Oh & Schuler, 2023a, 2023b). We see this general pattern in models trained on the Wikipedia dataset, which show more human-like agreement behavior than models trained on WSJ in our simulation of Bock and Cutting (1992).

In addition to size, we hypothesized that the training dataset can influence the model’s agreement behavior primarily by exposing the model to various agreement-related syntactic configurations. In particular, we hypothesized that greater exposure to these configurations will lead to more human-like behavior for simulations that rely on properties of those configurations (for example, models will process relative clauses better if they see more relative clauses during training). To test this empirically, we estimated the frequency of a number of relevant agreement configurations (subject-verb relations, relative clauses, disjunct subjects, etc.) for each of our simulations within the WSJ corpus as well as a subset of 500,000 sentences from the Wikipedia corpus. We parsed each sample of sentences from each corpus using the Chen and Manning (2014) dependency parser, and checked each resulting parse for each of the relevant syntactic configurations. The resulting counts are displayed in Table 2. Note that since the counts were derived from the output of an automatic parser, which may contain errors, they serve only as approximate estimates of the relevant frequencies.

**Table 2.** Counts of relevant syntactic phenomena in the WSJ Corpus and a subset of Wikipedia. Number-marked agreement relations are those in which a clear number feature is tagged by the parser for both the head of the subject and verb, and thus can teach the models about agreement. This is not the case in, for instance, the English past tense, where verbs are not marked for number (*the dogs barked* and *the dog barked* are both grammatical).

	WSJ		Wikipedia	
	Count	Per sentence	Count	Per sentence
Sentences	42068	1	500000	1
Subject-Verb relations	64694	1.54	658173	1.32
Number-marked agreement relations	17421	0.41	134362	0.27
RC subject modifiers	1427	0.034	8963	0.018
PP subject modifiers	7519	0.18	76708	0.15
Nested PP subject modifiers	1027	0.024	10091	0.020
Disjunct subjects	96	0.0023	1746	0.0035

One of the largest differences in structural frequency between the two corpora is in the case of disjunct subjects. We see a higher frequency of disjunct subjects in the Wikipedia corpus than in the WSJ Corpus, suggesting that the WSJ Corpus models’ human-like performance in our simulation of Haskell and MacDonald (2005) is not due to more extensive exposure to this construction. Instead, it could be that greater exposure to disjunct subjects leads to more hierarchical representations of disjunct subjects, reflecting the fact that the ordering of disjuncts is unimportant to the interpretation of the sentence. This would, in turn, lead to more consistent verb number responses regardless of the plural disjunct’s position: Since the ordering of disjuncts is more weakly encoded, ordering is less able to influence verb number. This insensitivity to ordering is in contrast with that of humans, who are biased towards the number of the closer disjunct (Haskell & MacDonald, 2005). The models’ behavior is consistent with traditional structural accounts of coordination where both disjuncts are assumed to be in a symmetric relationship, and as such linear position is irrelevant for operations like agreement (e.g., Williams, 1978). By contrast, a more linear representation of disjunction would lead to more uncertainty as to the number of the verb the model chooses to predict, leading to predictions that vary more severely when the ordering of disjuncts is swapped.

The one other notable difference across datasets concerns RCs, which are involved in the other simulation in which the Wikipedia-trained and WSJ-trained models differ in behavior (the simulation of the PP/RC asymmetry in Bock & Cutting, 1992). This suggests that our models, syntactic behavior is, in fact, affected by the differences in structural frequency between corpora of different genres. Given this pattern of construction frequency impacting syntactic processing behavior, if we aim to replicate the learning conditions of humans, we must acknowledge that the style of Wikipedia and the Wall Street Journal (i.e., formal and edited written text) is likely far different in distribution from what is typical of spoken language or child-directed speech. We will return to this point in the General Discussion.

#### What Improvements Does GPT-2 Show Relative to LSTM Models?

We compared our LSTM-based models (LM-Only and LM+CCG) to GPT-2, a much larger and more powerful language model. (See Table 3) GPT-2 differs from our models in multiple ways: the number of training samples, the number of learned parameters weights, and the models’ architectures. As such, it is difficult to draw conclusions about the sources of the differences in behavior between the GPT-2 and each of our models. We can, however, use GPT-2 to address other questions. In the present work we prioritized an investigation of the *qualitative patterns* of errors, but a long-term goal of this research program is arguably to also provide a quantitative match to human error patterns. If neural networks’ overall agreement error rates are uniformly much higher than those of humans, this goal is unlikely to be met. Using the stronger GPT-2 model we can ask, first, whether the LSTM models’ high rate of agreement errors is specific to these models, or whether it is a property of neural networks more broadly; and second, if GPT-2’s overall error rates are indeed lower, we can ask if there is there a relationship between overall error rates and the qualitative match between model and human error patterns.

**Table 3.** A summary of the experiments we simulated and the effects we found within LM-Only models, LM+CCG models and GPT-2. Each column represents whether we found the indicated effect in our simulations.

Effect in Humans	LM-Only	LM+CCG	GPT-2
Bock and Cutting (1992)			
PP > RC	✗	✗	✗
Number Asymmetry	✓	✓	✓
Franck et al. (2002)			
Syntactic Distance > Linear Distance	✗*	✗*	✓
Haskell and MacDonald (2005)			
Linear Distance	✓	✓	✓
Humphreys and Bock (2005)			
Notional Number	✗	✗	✗
Parker and An (2018)			
Core vs Oblique Arguments	✗	✗	✓
Attraction in Grammatical Sentences	✓	✓	✓
Wagers et al. (2009)			
Attraction from outside of RC	✓	✓	✗
Grammaticality Asymmetry	✓	✓	✗

* An effect is found in the LM-Only simulation of Franck et al. (2002), but in direction opposite of the effect found in humans.

In the PP vs. RC experiment of Bock and Cutting (1992) and the syntactic distance experiment of Franck et al. (2002), GPT-2 did in fact exhibit overall error rates comparable to humans. This indicates that the failure of our models to reach comparable overall error rates is due not to a fundamental issue with neural network models broadly.

This leads us to our second question: do more powerful models like GPT-2 always have more human-like error patterns? While this is the outcome we would expect if better overall agreement accuracy was highly correlated with human-like error patterns, the empirical answer to this question appears to be no. In our simulations of Bock and Cutting (1992), Haskell and MacDonald (2005), and Humphreys and Bock (2005), GPT-2’s errors did not match the human error pattern any more than the LSTM-based models did; worse, in our simulation of Wagers et al. (2009), GPT-2 failed to show the grammaticality asymmetry we found in all of our LSTM-based models. At the same time, the error patterns in the remaining two experiments did match the human one more closely. In our simulation of Franck et al. (2002), GPT-2 showed greater attraction effects from syntactically closer attractors than linearly closer ones; and in our simulation of Parker and An (2018), attraction effects were greatly attenuated when attractors appeared in core arguments compared to oblique ones. We see these differences as worthy of further investigation, particularly in light of accounts comparing the mechanisms of transformer-based models such as GPT-2 and the cue-based models of memory retrieval that are posited as explanations of Parker and An’s (2018) findings (Merkx & Frank, 2021; Ryu & Lewis, 2021; Timkey & Linzen, 2023).

Overall, we find that models with better overall syntactic competence and language modeling performance are not necessarily better matches to human behavioral patterns. This is convergent with prior work indicating that language modeling ability does not predict scores on syntactic benchmarks (Hu et al., 2020) and that performance on those syntactic benchmarks does not correlate with models’ ability to predict human behavioral measures like reading times or eye-movements (Wilcox et al., 2020). The relationship between language modeling performance and match to human behavioral patterns, however, is still unclear: some work finds that better language models are better matches to human behavior (Merkx & Frank, 2021; Wilcox et al., 2020), but others find the inverse relationship (Oh & Schuler, 2023b), with recent work suggesting a tipping point where improvements in language modeling reduce fit to human behavior (Oh & Schuler, 2023a). Given the size and training data available to our models, however, we believe that we are operating far before the tipping point Oh and Schuler (2023b) observed. Given this, our evaluation of human error behavior seems to run counter to prior results: We would expect to see that GPT-2 (the better language model) is significantly more human-like than LSTMs, but we find no evidence of this. One explanation of this discrepancy may lie in the difference in the kind of human behavior we and Oh and Schuler (2023b) seek to account for: While Oh and Schuler (2023b) attempt to explain broad-coverage human reading times, we attempt to explain patterns of agreement errors in particular.

## GENERAL DISCUSSION

In this paper we have proposed a framework for employing neural networks as broad-coverage models of human syntactic processing, and have used this framework to compare the errors made by humans in a suite of studies from the English subject-verb agreement processing literature to the errors made by two classes of networks based on the LSTM architecture: first, LM-Only models, which were trained solely on word prediction over a text corpus; and second, LM+CCG models, which were trained on word prediction as well as CCG supertagging, a task that requires sophisticated representations of syntactic relationships between words, and thus, we reasoned, should share those sophisticated syntactic representations with the word prediction component.

Both classes of models successfully simulated some human results, but failed to simulate others. They were especially unsuccessful in replicating human error patterns that can be attributed to syntactic structure; contrary to our hypothesis, LM+CCG models did not show more sophisticated, human-like syntactic performance than LM-Only models, although they did perform in a more human-like manner than LM-Only models in some of the simulations that were not directly linked to syntactic structure. Follow-up analyses indicated that training on CCG supertagging may not have required models to learn more sophisticated syntactic representations than learned from next word prediction alone.

We also assessed the sensitivity of our results to the training corpus by repeating a subset of our simulations using models with the same architecture as before trained only on 80 million words of English Wikipedia, or only on the approximately one million words of the WSJ Corpus. Models trained on Wikipedia did not consistently exhibit more or less human-like syntactic behavior than models trained only on the much smaller WSJ Corpus subset. However, we do find that the frequency of the relevant syntactic constructions in each corpus can explain the differences in agreement behavior. We take this to indicate that the behaviors our models learn are sensitive to training set size and style.

In the sections below, we will discuss these findings and their implications more broadly. We will then consider the potential for the use of neural network language models as cognitive models of the processing of syntactic constraints like agreement, as well as the possible pitfalls and best practices that emerge from our experiments.

### Does Adding a Pressure Toward Sophisticated Syntactic Representations Lead to More Human-Like Syntactic Performance?

As discussed earlier, our experimental results (summarized in Table 4) suggest that the syntactic information used for CCG supertagging only affects agreement attraction patterns modestly, and, counter to our hypotheses, does not help models simulate human behavior in syntactically complex environments. In this section, we will discuss both why supertagging did not impact our models in the way we expected, as well as how we could build models that better capture the syntactic factors modulating agreement processing.

**Table 4.** A summary of the experiments we simulated using LM-Only and LM+CCG models. The LM-Only column indicates whether LM-Only models displayed a significant effect in the same direction as the original studies’ authors found, and the LM+CCG column indicates whether we found a significant interaction between the relevant effect and the addition of CCG supertagging training, as well as the direction of that interaction.

Effect in Humans	LM-Only	LM+CCG	LM+CCG More Human-like?
Bock and Cutting (1992)			
PP > RC	✗	No Difference	
Number Asymmetry	✓	Larger Effect	✓
Franck et al. (2002)			
Syntactic Distance > Linear Distance	✗*	No Difference	
Haskell and MacDonald (2005)			
Linear Distance	✓	Larger Effect	✓
Humphreys and Bock (2005)			
Notional Number	✗	No Difference	
Parker and An (2018)			
Core vs Oblique Arguments	✗	No Difference	
Attraction in Grammatical Sentences	✓	Smaller Effect	✓
Wagers et al. (2009)			
Attraction from outside of RC	✓	No Difference	
Grammaticality Asymmetry	✓	No Difference	

* An effect is found in the LM-Only simulation of Franck et al. (2002), but in direction opposite of the effect found in humans.

#### Why Didn’t Supertagging Lead to Better Simulations of Syntactic Experiments?

The error patterns corresponding to the contrasts that are most closely tied to syntactic structure—PP vs. RC (Bock & Cutting, 1992) and linear vs. syntactic distance (Franck et al., 2002)—were not more human-like in LM+CCG than LM-Only. We hypothesized that one potential explanation may be that the representations models’ learned during training on CCG supertagging were not those recruited for word prediction during evaluation. To test this, we conducted two analyses to determine whether the parts of our models’ representations that are used for supertagging are necessary for our models’ word prediction performance.

The results of these two analyses present a mixed picture. Our ablation analysis found that neurons in LM+CCG models whose removal impacted supertagging performance were also important for word prediction performance, suggesting that representations between tasks overlap significantly. Our amnesic probing analysis, which considered the possibility of distributed representations of syntactic structure, found that removing information useful for supertagging led to a sharp decrease in LM+CCG models’ word prediction ability, but, crucially, found a similar amount of information useful to supertagging in LM-Only models; erasure of that information led to similar decrease in word prediction performance as for LM+CCG models. This suggests that all of the information used for CCG supertagging may emerge from the model’s language modeling component. This recontextualizes the ablation analysis: representations important for supertagging and language modeling are shared only insofar as language modeling representations are sufficient for both tasks.

These results, taken together, point toward the inadequacy of CCG supertagging as an auxiliary task for improving the syntactic representations of even simple LSTM language models without explicit syntactic inductive biases. Multi-task training on both word prediction and CCG supertagging fails to create more sophisticated syntactic representations, both in terms of match to human behavior (on the explicitly syntactic agreement phenomena) and in terms of the performance of supertagging classifiers that use those representations.

While the auxiliary syntactic objective did not make performance more human-like across the board, it also did not make performance *less* human-like. In each case, performance either did not change significantly or, in three cases, became more human-like. We take this as evidence that the more human-like behavior of LM+CCG models is not due just to random variation in the optimization process: if that was the case case we would expect changes in either direction with equal likelihood. Thus, despite a lack of significant changes in LM+CCG models’ behavior on the specific, explicitly syntactic tasks we simulated, this pattern of results is consistent with the claim that additional pressure for models to represent syntactic properties of their input leads to more human-like behavior broadly.

#### How Can We Create Models With More Human-Like Syntactic Processing?

Auxiliary training objectives are, at least in principle, an attractive tool, for a number of reasons: they can be implemented with minimal modification to model architecture; we can verify that the model has encoded the relevant information by monitoring its performance on the objective; and the idea that the representations used in language processing are shaped by the competing needs of various linguistic tasks is cognitively plausible (see, for example, the influence of orthographic pressures on the phonological representations used to detect rhymes, Seidenberg & Tanenhaus, 1979). Our negative results suggest, however, that auxiliary training objectives, or at least the CCG supertagging objective we used, may not be a sufficiently effective tool for aligning the syntactic processing behavior of neural networks and humans.

How can we create models whose agreement error patterns show a human-like sensitivity to hierarchical structure? One potential path forward is to increase the sophistication of the syntactic structures that models are pressured to learn. CCG supertagging primarily requires sensitivity to local syntactic structure (i.e., as represented in the way a word combines with adjacent constituents). Models could become more sensitive to larger syntactic context through pressures to construct incremental representations of parse states: Qian et al. (2021), for instance, found that models trained to generate parser action sequences were more successful on syntactic benchmarks than those trained on word prediction and an auxiliary syntactic task (specifically, predicting a window of parser actions that would occur around the parsing of the current word).

We can also change the auxiliary task by varying syntactic formalism we use to generate the representations we pressure models to learn. Other syntactic formalisms such as Minimalist Grammars (Stabler, 1997) or Tree-Adjoining Grammars (Joshi et al., 1975) may encode syntactic constraints in a manner that better reflects human processing.

As an alternative approach, we could abandon auxiliary training objectives altogether and, instead, consider architectures that condition word prediction more directly on syntactic representations. The Recurrent Neural Network Grammar (Dyer et al., 2016) architecture, for example, acts as a language model, but constructs explicit syntactic parses of its input during processing. This structure encourages the model to learn how best to use the hierarchical information contained in those parses to predict upcoming words. Prior work evaluating the syntactic abilities of these models have found them to be substantially better than LSTMs at predicting measures of processing difficulty in humans (Hale et al., 2018), and, again, objectives related to modeling parsing explicitly have been shown to lead to better performance on syntactic benchmarks than auxilliary tasks (Qian et al., 2021).

Transformer architectures (Vaswani et al., 2017), like the GPT-2 model we evaluated, have also displayed significantly stronger syntactic abilities than LSTMs, particularly when trained on very large datasets (Hu et al., 2020). Transformer-based models have also been argued to implement processes akin to cue-based memory retrieval (Ryu & Lewis, 2021), a mechanism which is widely used to explain phenomena in agreement processing, as well as sentence processing more broadly (Badecker & Kuminiak, 2007; Lewis et al., 2006; Parker & An, 2018; Wagers et al., 2009). While our simulations using the transformer-based GPT-2 did not produce error patterns substantially closer to humans than LSTMs, we only explored a single transformer model, and thus a more thorough investigation of transformers—and the inductive biases inherent to that architecture—may show promise. At the very least, transformers such as GPT-2 obtain lower overall error rates than the LSTMs we trained.

### Do the Models Learn Similar Syntactic Behavior From Different Types of Training Data?

In our training data experiments (results summarized in Table 5), we found that models trained solely on Wikipedia exhibited more human-like agreement error patterns when tested on PP and RC attractors than those trained on the WSJ Corpus. We also found that models trained on the WSJ Corpus agreed with the closer disjunct much more often than models trained on Wikipedia; in this respect the WSJ Corpus models were closer to human behavior. This pair of findings indicates that models’ syntactic processing behavior, as measured by their error patterns, is sensitive to differences in the size and genre of the models’ training corpus.

**Table 5.** A summary of the experiments we simulated and the effects we found within LM-Only models trained solely on Wikipedia and solely on the Wall Street Journal portion of the WSJ Corpus.

Effect in Humans	LM-Only Wiki + WSJ	LM-Only Wiki	LM-Only WSJ
Bock and Cutting (1992)			
PP > RC	✗	✗	✗*
Number Asymmetry	✓	✓	✗
Franck et al. (2002)			
Syntactic Distance > Linear Distance	✗*	✗*	✗*
Haskell and MacDonald (2005)			
Linear Distance	✓	✓	✓

* An effect is found, but in the opposite direction from humans.

For the purposes of using neural network language models as cognitive models, this sensitivity to small perturbations in training data is potentially worrying: if models are not sufficiently robust to variation in training data, the particular composition of the training dataset becomes a critical part of our cognitive model’s assumptions. The English Wikipedia corpus, though representative of a particular variant of English, is not representative of the data observed either by a child acquiring language or by the average native speaker. This is also true of the WSJ Corpus, which is composed primarily of financial news articles. There are two major approaches we can take to address this problem: first, we could ensure that models trained for the purposes of cognitive modeling are trained on datasets that closely approximate a child’s input (i.e., the CHILDES child-directed speech corpus; MacWhinney, 2000; Yedetore et al., 2023). Alternatively, we could build models with stronger inductive biases that aim to limit the amount of variation that can be caused by the input data. While the supertagging objective may have weakly constrained the types of solutions our models could find during training, stronger architectural inductive biases, like those imposed in models like Recurrent Neural Network Grammars (Dyer et al., 2016), may increase robustness to variation in training data.

### Which Linking Function Should We Use to Model Agreement Processing?

To turn neural network models into psycholinguistic models of agreement processing in production, we needed a to convert the model’s output to a format that is comparable to the results of human sentence completion experiments. Two approaches to this problem that are distinct from the one-sample linking function we described in the Methods section appear in prior work. Here we contrast our method with these alternatives and provide a psycholinguistic interpretation of one class of potential linking hypotheses.

Linzen and Leonard (2018) sidestep this problem altogether by training their neural network as a verb number classifier: the decoder directly predicts the number feature of the verb from the preamble. This technique has two major limitations. First, it requires training data that is annotated with the number and position of the verb. From a cognitive perspective, such annotations are unlikely to be available to human learners; from a practical perspective, it is very costly to produce these annotations manually, and unreliable to do so automatically. The second limitation is that this training method prevents the model from learning syntactic constraints other than agreement, which could be used to better predict agreement patterns. This contrasts with language models, which are incentivized to build representations for any property that might help them predict the next word. Those representations are available to the model when it predicts the verb, and thus the verb’s number. By contrast, the only training signal available to a number classifier is whether or not it predicts the following verb’s number correctly, and thus such a model is not incentivized to build representations for any other linguistic properties, including those that might interact with agreement in agreement attraction contexts.

Another common approach was introduced by Linzen et al. (2016), which we will refer to as max-prob. Like our method, max-prob attempts to convert the probabilistic next-word predictions of a language model to agreement behavior. Under this paradigm, a candidate pair of the singular and plural forms of a verb is selected, and the probabilities assigned by the language model to the two forms are compared. The model is evaluated as if it had produced the form whose probability is higher, regardless of the magnitude of the difference between the probabilities of the two forms.

The one-sample method we use preserves certain features of max-prob. Like max-prob, one-sample selects a candidate singular/plural pair of verbs (e.g., “write” and “writes”) prior to the selection of the verb’s number feature. This design choice can be seen as reflecting two sequential stages posited by some theories of language production (Bock & Levelt, 1994; Levelt et al., 1999): first, lemma selection—the selection of the word’s canonical, morphologically unmarked form; and second, grammatical encoding, where grammatical features, like number, are marked. Under this interpretation, the model plus linking function combination presented here aims to capture only the second stage: grammatical encoding.

The main difference between max-prob and one-sample is that one-sample selects the output form probabilistically, with the probability of a singular form proportional to the probability assigned to the singular candidate by the language model. This gives one-sample one major advantage over max-prob: it is sensitive to differences in language model probabilities between the singular and plural verb forms, thereby capturing subtle effects that would be obscured if we used the max-prob linking function.

Another consequence of using one-sample is that our models exhibit non-deterministic behavior for a particular experimental item. Under max-prob, a model that assigned a probability of 51% to the grammatical form would be taken to consistently produce the correct form of the verb. By contrast, under one-sample such a model would be only slightly above chance at producing the grammatical form of the verb. This is true even when the margin between the correct and incorrect forms’ probabilities is large: a model that assigns 80% probability to the grammatical form would still produce errors in one out of five simulated trials when given the same preamble. This stochasticity better reflects the non-deterministic nature of human agreement errors—we would not expect a participant to always or never make errors on a particular item, but rather make an error on that item with some probability.

The difference between max-prob and one-sample can be viewed as a reflection of the competence-performance distinction (Chomsky, 1965). The goal of max-prob-based analyses is to determine whether a model has acquired the linguistic *competence* of subject-verb agreement (i.e., that the verb should agree with the subject in number). By contrast, our goal is to construct a model that makes the same errors in *performance* as humans. Thus we use our one-sample method, which models production of a verb as drawing a sample from the probability distribution provided by a language model, rather than the max-prob method. These two linking hypotheses lie at two ends of a spectrum of potential modeling assumptions: under a paradigm where we take *n* samples from the distribution over the candidate pair provided by our language model and select the form sampled most often, one-sample is the case where we are limited to a single sample, while max-prob matches the behavior in the limit as *n* approaches infinity. Future work might explore fitting *n* to human data, or comparing various choices of *n* to human behavior under various degrees of time pressure or memory load. For instance, one might expect that under high time pressure, human behavior might match an *n* closer to 1, while in an untimed proofreading task, behavior might match much higher values of *n*.

Modifications to one-sample may also help bring our models’ error rates more in line with that of humans. Models based on one-sample will often assign significant probability mass to the form of the verb that the language model judges as less likely, which results in the high agreement error rates we observe in our simulations. This contrasts with max-prob models, which assign no probability mass to the less likely form and thus, as discussed above, are insensitive to the underlying language model’s level of certainty. Selecting a linking hypothesis that lies between these two extremes may lead to the best of both worlds, simultaneously preserving one-sample’s sensitivity and reducing the overall rate of agreement errors. We leave an investigation of alternative linking functions for future work.

### What Can Neural Networks Contribute to the Study of Human Syntactic Processing?

Most psycholinguistic modeling, including in the area of agreement processing, adopts a cognitive process modeling approach—models are hand constructed, and consist of a number of interpretable, primitive cognitive operations organized sequentially (Gregg & Simon, 1967); each of these operations may have a small number of parameters that are fit to behavioral data. These models have, as their primary benefit, the ability to implement specific psycholinguistic hypotheses about the phenomena in question.

By contrast, neural networks are, on their face, black boxes (McCloskey, 1991). While we can attempt to modulate their behavior by changing their architecture and training task (or tasks), the mechanisms implemented by the model are learned from data during training. For psycholinguists, this is a double-edged sword: it prevents us from testing a specific algorithmic theory like we could with a cognitive process model, but it also allows the model to develop solutions that one may not have otherwise considered. This ability to learn potentially novel solutions from data allows neural network models to be used to evaluate claims in terms of relevant inductive biases or learning pressures. In this work, we asked whether adding explicit pressure toward more sophisticated syntactic representations would lead models to make more human-like agreement errors. By comparing models with and without that additional pressure, we could address that question, and determine whether strong syntactic representations were sufficient to explain the human patterns of agreement errors. Crucially, this was done without committing to a particular agreement mechanism, and without losing broad coverage: both types of models could be used to simulate agreement in any construction.

Another benefit of neural network modeling is that the mechanisms employed by neural networks are necessarily *learnable* solutions; if our training task is ecologically valid, and our data is comparable to data a human might be exposed to, any solution developed by the model is, given the inductive biases assumed by our model choice, learnable from the input (Rumelhart & McClelland, 1987, among others). This is in contrast to traditional cognitive process models, where it is often unclear how humans come to possess the hypothesized mechanism.

The particular learning objective we use involves predicting the next word over large natural corpora. Given the wealth of evidence that humans do something akin to word prediction during sentence processing (for a review, see Kutas et al., 2011), we take word prediction as a reasonable choice of training task (Elman, 1990). Our training data does, however, present two issues that complicate the analogy to human learning. First, the type of corpora we used—encyclopedia or newspaper articles—are not comparable to the input that children have access to when acquiring language, though they do roughly match the quantity of children’s input: in the tens of millions of words. Future work attempting to strengthen the learning argument could consider using corpora of child-directed speech (i.e., CHILDES, MacWhinney, 2000) to evaluate whether less linguistically complex training data leads to similar behavior (Yedetore et al., 2023). The second issue is that we must ensure that the amount of the data our models receive is comparable to that needed by humans to achieve a similar set of behaviors. In the long term, this perspective suggests considering all processing phenomena from the perspective of acquisition: can we construct a model that captures the relevant phenomena at the same stage of “acquisition" as human children?

Learnability considerations aside, a critic may still argue that the syntactic processing mechanisms that models like ours learn are still insufficiently *explanatory*. Because the model’s predictions are generated by a series of ostensibly uninterpretable matrix operations, referring to a neural network model as a model of language processing is merely replacing one black box—a human participant—with another—a neural network. That is, while neural network models can act as instantiations of broad cognitive principles (i.e., prediction; Goldstein et al., 2022), a critic may argue that those principles are too coarse to act as a proper mechanistic theory. We believe that this problem is not insurmountable. Unlike human participants, the inner workings of a neural network model can be recorded, probed, ablated, and inspected in a variety of other ways with little difficulty and without ethical concerns, allowing researchers to approximate high-level, more easily interpretable operations that are implemented by a particular neural network (see, for example, Elazar et al., 2021; Finlayson et al., 2021; Hupkes et al., 2018; Lakretz et al., 2019; Ravfogel et al., 2021). While mechanistic explanations of processing do not come for free from neural network models, as they do in more traditional psycholinguistic models, the fact that its possible to analyze their internal computations lends them some transparency.

We began by asking what behavior a simple linear sequence learner with no explicit syntactic pressure toward hierarchical syntactic representations exhibits after being trained on word prediction. We then compared this model’s agreement error patterns to a model with an explicit syntactic training objective. Continuing to pursue this approach by analyzing models with stronger and stronger pressures toward sophisticated syntactic representations allows for a bottom-up approach to understanding phenomena like agreement attraction parallel to traditional hypothesis building. First, through this exploration in the hypothesis space, we find the right biases and pressures sufficient for neural models to capture human performance, and then construct specific mechanistic hypotheses about the cognitive processes that give rise to particular behavioral phenomena using neural network analysis techniques. These mechanistic hypotheses then serve to connect the particular innate or external biases and constraints that characterized our neural network model with traditional psycholinguistic models of the representations and processes that govern language processing.

### How Do Our Results Bear on Existing Accounts of Agreement Attraction?

As discussed in the previous section, we see our neural network modeling approach as complementary to existing symbolic models of agreement attraction errors, and in this work we have sought to model a set of experiments from the literature that motivate a number of existing symbolic approaches to explaining agreement errors. In this section, we will focus on how the results of our experiments relate to two accounts of agreement errors, feature percolation and retrieval interference.

*Feature Percolation* accounts of agreement attraction (Franck et al., 2002, etc.) propose that agreement errors are fundamentally encoding errors: they emerge when the speaker or reader erroneously encodes the wrong number feature on the subject. More specifically, they propose that in sentences that exhibit agreement attraction from subject modifiers, the number feature from a noun in the modifier “percolates” upward through the sentence’s hierarchical structure to the level of the subject. This contrast with the correct processing of agreement, where it is the number feature of the head of the subject that is expected to percolate to this level. Crucially, these proposals suggest that attraction errors are sensitive to a sentence’s syntactic structure: the rate of attraction errors is expected to be inversely proportional to how far a feature needs to erroneously percolate to cause an attraction error. The experiments from Bock and Cutting (1992) and Franck et al. (2002) we simulated provide evidence for this account: they demonstrate that the syntactic distance between the subject and attractor affects agreement attraction error rates in humans. We find that both our LM-Only and LM+CCG models can encode relatively sophisticated syntactic structure, as evidenced by the CCG supertagging accuracy of classifiers trained on their representations, but still fail to replicate the syntactic distance effects found in humans. These results corroborate the importance of tying agreement mechanisms to structural representations: Syntactic distance effects are not simply emergent from the presence of syntactic structure and pressure to learn agreement.

By contrast with the Bock and Cutting (1992) and Franck et al. (2002) experiments, which support the feature percolation accounts, the grammaticality asymmetry result from Wagers et al. (2009) points to the inadequacy of these accounts (though see Hammerly et al., 2019). Wagers et al. (2009) instead argue for a *retrieval interference* model of agreement errors, where agreement errors emerge not from an error in encoding, but rather an error in retrieving the number feature of the subject when the agreement computation is conducted at the verb. Typically, these accounts rely on cue-based retrieval models of memory to predict the frequency of retrieval errors that lead to agreement attraction errors (Badecker & Kuminiak, 2007; Wagers et al., 2009, etc.). Our results demonstrate that the results Wagers et al. (2009) found are derivable from LSTMs, suggesting that the encoding-decoding scheme learned by these models represents an alternative or equivalent approach to cue-based retrieval for explaining grammaticality asymmetry effects. Exploration of the encoding schemes used by these models may shed light on alternative accounts of these effects: Lakretz et al. (2019, 2021) find that LSTM models similar to ours encode number features in a dense, localized manner. These models often encoded number for multiple noun phrases in embedded structures (like those used in Wagers et al., 2009) in a single dimension of the model’s representations, leading to lossy encodings of number whose decoding/retrieval may look fairly different from that in cue-based models.

Rather than seeking a neural network alternative to cue-based accounts, Ryu and Lewis (2021) find that the attention mechanisms in models like GPT-2 may implement some principle of cue-based retrieval. Work into comparing the encoding and retrieval mechanisms employed by different neural architectures (i.e., Timkey & Linzen, 2023) may serve as fertile ground for exploring the hypothesis space consistent with results like Wagers et al.’s (2009) grammaticality asymmetry.

Of course, encoding and retrieval based accounts of agreement attraction are not mutually exclusive. For example, Yadav et al. (2023) find that hybrid models, where errors can be due to either encoding or retrieval, predict human agreement errors better than non-hybrid models. In this sense, our approach can also be seen as a hybrid model, as errors can arise in either stage.

## CONCLUSION

In this paper, we have proposed a framework for using neural language models to model human syntactic processing, and used that framework to evaluate the ability of a variety of neural language models with different training data and training objectives to simulate results from the agreement attraction literature. We aim to answer three questions: what behaviors arise in LM-Only models, which are trained just to predict the next word? Do LM+CCG models, which are provided with explicit syntactic supervision, perform in a more human-like way? Does the size and genre of the models’ training corpus influence syntactic behavior?

Our simulations leave us with a few key findings: (1) neural network language models can capture a number of syntactic agreement effects, including linear distance effects, the grammaticality asymmetry and the number asymmetry; (2) much of the syntactic information a model must learn for an auxiliary syntactic task may already be learned from word prediction; and (3) the ability of a language model to capture agreement phenomena is dependent not only on the inductive biases imbued by the models’ architecture and pressure from training objectives, but also the size and composition of its training data.

More broadly, we see this work as the first step in constructing a neural network-based approach to modeling and understanding online agreement processing, and human syntactic processing more broadly. Under this approach, we first characterize the biases and pressures necessary for matching human performance, then analyze the behavior and internal representations of such human-like models to generate detailed and testable hypotheses to be tested in humans. Crucially, this “bottom-up" approach is complementary to the cognitive process modeling approaches that are currently standard in psycholinguistics. The issues inherent in cognitive process modeling—determining the learnability of a particular account, as well as determining the breadth of the empirical phenomena that account covers—can be addressed by using neural network approaches to generate and test statistically learned hypotheses. The work presented here works toward completing the first stage, helping characterize the biases and pressures on learned representations necessary to match human syntactic processing and evaluating a method for imbuing models with one such bias.

## ACKNOWLEDGMENTS

The authors thank Colin Wilson, members of the NYU Computation and Psycholinguistics (CAP) Lab, and the Society for Human Sentence Processing (HSP) community for valuable feedback and discussion.

## FUNDING INFORMATION

This work was supported in part through the NYU IT High Performance Computing resources, services, and staff expertise. This work was additionally supported by the United States–Israel Binational Science Foundation (award no. 2018284).

## AUTHOR CONTRIBUTIONS

S.A.: Conceptualization; Investigation; Methodology; Software; Visualization; Writing - original draft. T.L.: Conceptualization; Funding acquisition; Supervision; Writing - review & editing.

## DATA AVAILABILITY STATEMENT

Code and simulation data from all of our our analyses is available at https://github.com/SArehalli/NNSCognitiveModelsAgreement.

## Notes

^1^ Assigning probabilities to strings of words and providing a distribution over the next word in a sequence are equivalent, since *P*(*w*_1_ … *w*_*n*_) = *P*(*w*_1_)*P*(*w*_2_ | *w*_1_)*P*(*w*_3_ | *w*_1_, …, *w*_2_) … *P*(*w*_*n*_ | *w*_1_, …, *w*_*n*_) for words *w*_1_, …, *w*_*n*_.^2^ Since perplexities are sensitive to tokenization choices, it is difficult to compare perplexities across different training set-ups to assess how well-trained a particular model is. Since model perplexities are very similar across different instances of our models, we provide the top predictions of one model for sample preambles in Appendix B to demonstrate what our model has learned during training.^3^ We use the term “cognitive model” here only to distinguish the models we create, which aim to predict human experimental measures like error rates and reading times, from the language models that underlie them, which aim only to predict the next word. While our eventual goal is to use our cognitive models to investigate the cognitive processes that generate those experimental measures, we do not use the term here to indicate that these models provide an explicit, interpretable account of a particular human cognitive process. See the General Discussion for a further discussion of how these models relate to the more traditional cognitive models used in psycholinguistics.^4^ Note that while this is true in many syntactic analyses (Gazdar et al., 1985; Jackendoff, 1977), including the one adopted by Haskell and MacDonald (2005), asymmetric analyses of coordination are common in minimalist approaches to syntax (i.e., Cormack & Smith, 2005; Kayne, 1994). That being said, in a standard asymmetric analysis (Kayne, 1994), the second disjunct forms a constituent with *or* and is thus more syntactic distant from the verb than the first disjunct. This means that linear and syntactic distance still make opposing predictions in Haskell and MacDonald’s (2005) materials.^5^ Analysis used the model formula *error_rate* ∼ *subj_num* * *attr_subj_match* * *pp_or_rc* * *log*(*freq*) + (1 | *model*) + (1 | *item*).

## Figures and Tables

**Figure A1.**
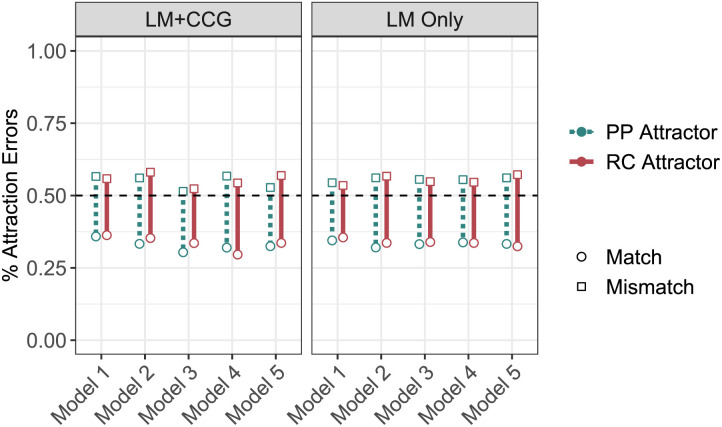
Error rates from our simulations of Bock and Cutting (1992) averaging over 557 singular and plural verb pairs extracted from the WSJ Corpus.

**Figure A2.**
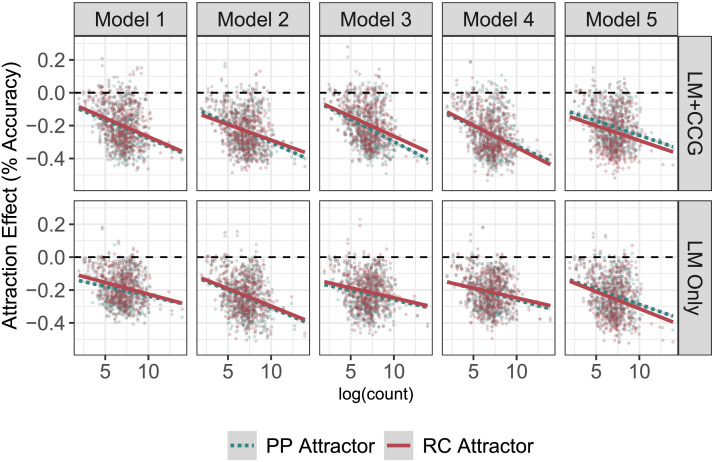
Agreement Attraction effects (Subject-Attractor Mismatch minus Match Error Rates) from our simulations of Bock and Cutting (1992) for each of the 557 singular and plural verb pairs extracted from the WSJ Corpus.

**Figure D1.**
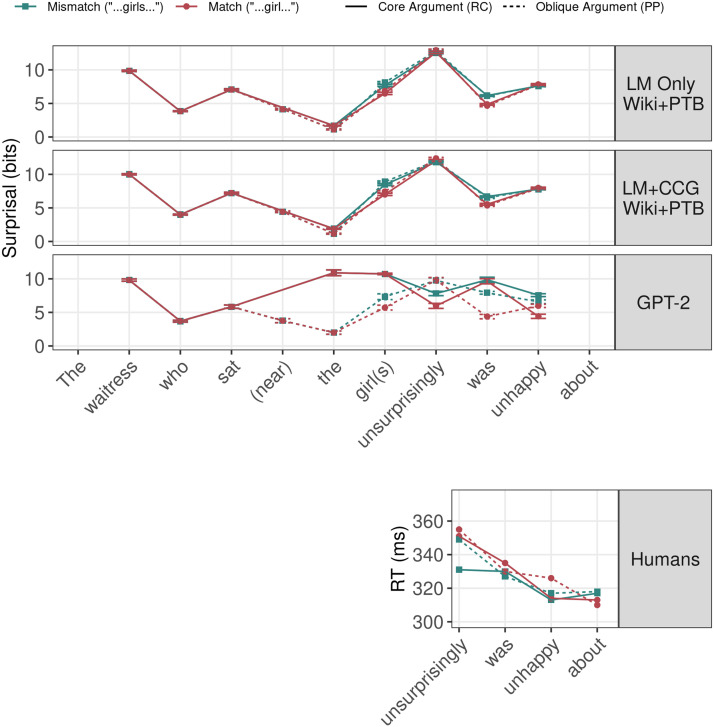
Word-by-word surprisals for models in our simulation of grammatical materials from Parker and An (2018). Error bars are standard errors. Since models were given no context prior to the first word, no surprisal is given for the first word of the sentence (*The*). Since *near* only appears in the oblique argument condition, no surprisal is provided for the token in the core argument condition. The critical region here is at the verb *was/were*, where the grammaticality of the agreement relation becomes clear. If an attraction effect manifests in grammatical sentences, surprisal will be higher in the mismatch condition than for those in the mismatch condition.

**Figure D2.**
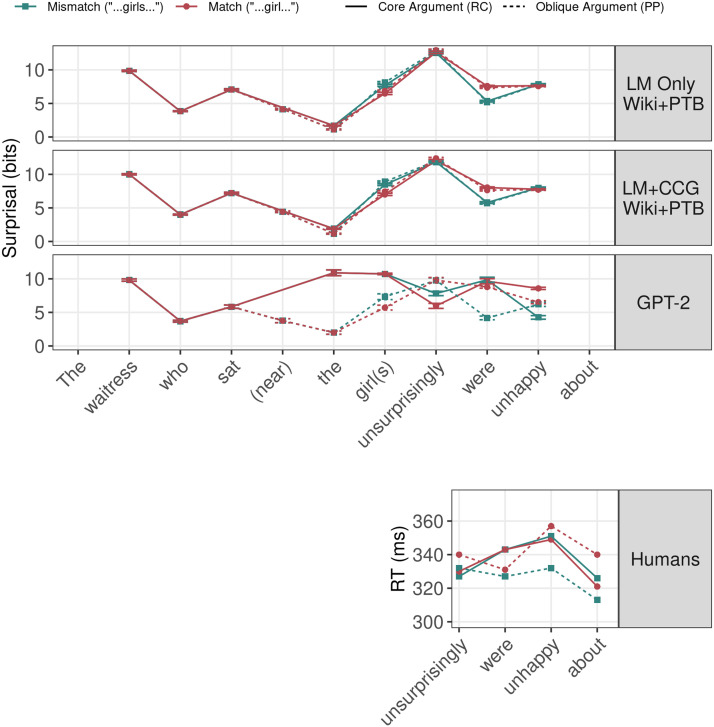
Word-by-word surprisals for models in our simulation of ungrammatical sentences from Parker and An (2018). Error bars are standard errors. Since models were given no context prior to the first word, no surprisal is given for the first word of the sentence (*The*). Since *near* only appears in the oblique argument condition, no surprisal is provided for the token in the core argument condition. The critical region here is at the verb *was*/*were*, where the grammaticality of the agreement relation becomes clear. If such an effect manifests in ungrammatical sentences, surprisal will be lower in the mismatch condition than in the match condition.

**Figure D3.**
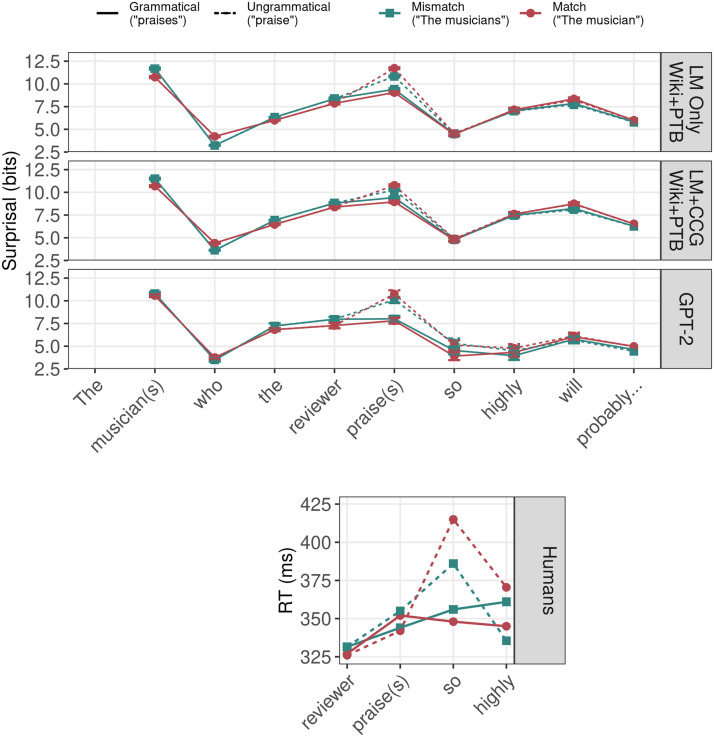
Word-by-word surprisals for models in our simulation of sentences with a singular subject from Wagers et al. (2009). Error bars are standard errors. Since models were given no context prior to the first word, no surprisal is given for the first word of the sentence (*The*). The critical region here is at the verb *praise(s)*, where the grammaticality of the agreement relation becomes clear. If an attraction effect manifests in grammatical sentences, surprisal will be higher in the mismatch condition than for those in the mismatch condition. If such an effect manifests in ungrammatical sentences, surprisal will be lower in the mismatch condition than in the match condition.

**Figure D4.**
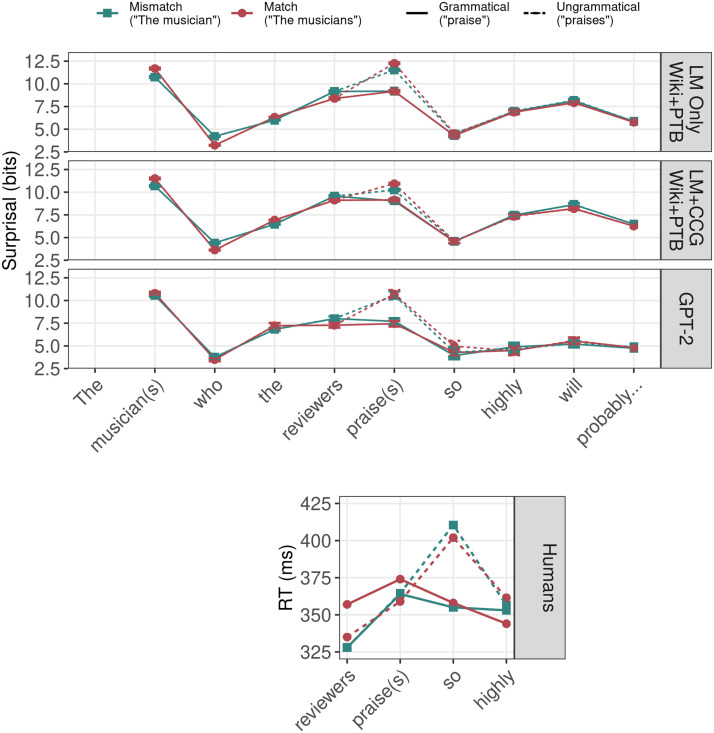
Word-by-word surprisals for models in our simulation of sentences with a plural subject from Wagers et al. (2009). Error bars are standard errors. Since models were given no context prior to the first word, no surprisal is given for the first word of the sentence (*The*). The critical region here is at the verb *praise(s)*, where the grammaticality of the agreement relation becomes clear. If an attraction effect manifests in grammatical sentences, surprisal will be higher in the mismatch condition than for those in the mismatch condition. If such an effect manifests in ungrammatical sentences, surprisal will be lower in the mismatch condition than in the match condition.

## APPENDIX A: THE EFFECT OF VERB CHOICE

In the simulations of production experiments in the main text, we derive the agreement error rate from the difference in probabilities our models assign to singular and plural forms of the word *be*. If our models are learning an abstract agreement mechanism (as opposed to a lexically specific mechanism), we should expect our results to generalize to other verbs. In this section, we evaluate that expectation in the case of our simulation of Bock and Cutting (1992).

To do this, we first collected a set of 557 pairs of singular and plural verb forms that appear in the Wall Street Journal portion of the Penn Treebank (Marcus et al., 1993), extracted based on their part-of-speech annotations. We then ran our simulation of Bock and Cutting (1992) using the probabilities of each of these singular and plural verb forms for each of our LM-Only and LM+CCG models trained over the full WSJ+Wikipedia training set. Results of this analysis averaged over all of these verbs are shown in Figure A1.

Part of our motivation for using forms of the verb *be* in our main analysis was a concern that singular and plural verb forms with lower frequency may not have their number features well represented in our models. Given this concern, we extracted the frequencies of the singular forms of our verbs from the Corpus of Contemporary American English (COCA; Davies, 2019). The attraction effect for each verb by verb frequency is shown in Figure A2.

A beta mixed-effects regression^5^ revealed a significant attraction effect (LM+CCG: *β* = −0.17, *z* = −7.89, *p* < 0.001; LM-Only: *β* = −0.36, *z* = −18.21, *p* < 0.001), but no significant interaction between the attraction effect and whether the modifier was a PP or RC (LM+CCG: *β* = −0.092, *z* = 1.19, *p* = 0.23; LM-Only: *β* = 0.049, *z* = 1.70, *p* = 0.09), matching the conclusions of the analysis in the main text: models do not capture the PP/RC asymmetry Bock and Cutting (1992) found in humans. We did find a significant interaction between the attraction effect and the log frequency of the candidate singular/plural verb pair we used to evaluate agreement, where evaluating with more frequent verbs led to greater attraction effects (LM+CCG: *β* = −0.092, *z* = −28.88; *p* < 0.001; LM-Only: *β* = −0.068, *z* = −23.34, *p* < 0.001). We also found a significant negative effect of log frequency on error rates (LM+CCG: *β* = −0.08; *z* = −38.01; *p* < 0.001; LM-Only: *β* = −0.05, *z* = −25.50, *p* < 0.001). These results are consistent with the hypothesis that lower frequency verbs have a less specified number in our models’ representations, and thus are less sensitive to agreement constraints and attraction phenomena. However, these results are also consistent with a hypothesis where the agreement mechanism in our models is sensitive to the agreeing verb’s frequency. We leave further investigation of these properties, as well as their implications for the modeling of human data, to future work. (See, for intstance, Wei et al., 2021.)

## APPENDIX B: SAMPLE MODEL PREDICTIONS

In this appendix, we provide the top 5 generations, along with their probabilities, from one of each of our two primary model classes: LM-Only and LM+CCG. Since model perplexities are difficult to compare given differences in vocabulary and test set, we provide these top-ranked continuations to allow for a qualitative evaluation of the word-prediction abilities of our models.

**Table B1.** Top-5 predictions and their log-probabilities from one of our LM+CCG models

Preamble	Prediction	Log-Probability
The key to the cabinets …	of	−1.34
	,	−2.49
	is	−2.83
	are	−3.40
	was	−3.53
The key to the cabinet …	of	−1.54
	is	−2.38
	’s	−2.68
	,	−3.03
	was	−3.17

**Table B2.** Top-5 predictions and their log-probabilities from one of our LM+CCG models

Preamble	Prediction	Log-Probability
The key to the cabinets …	was	−1.46
	of	−1.87
	is	−2.06
	,	−3.04
	and	−3.23
The key to the cabinet …	is	−0.99
	was	−1.68
	,	−3.11
	of	−3.14
	’s	−3.27

## APPENDIX C: EDITS TO EXPERIMENTAL ITEMS

The neural network models we train operate on the word level, and depend on the set of words contained in the models’ training sets in order to learn word-level representations. When a model encounters a word it has not seen in training, it uses the representation of a special <UNK> token that replaces words that appear fewer than five times in the corpus.

Because most experimental manipulations depend on the features of a particular word, the experimental materials we use in our simulations must be edited so as to avoid <UNK> tokens preventing the models’ from being able to interpret those features. Below, we will list, for each set of experimental materials, the changes made to those materials to match the vocabulary of the Wikipedia dataset. Due the the significant vocabulary limitations of the WSJ Corpus dataset, we provide a full list of the modified items. Since our goal is to replace rare words, which were excluded from the models’ vocabulary, with words that the models have observed, the frequency of the new words is necessarily higher than of the words they replace. We do not control for orthographic properties such as word length, since our LSTM models treat words as atomic units and thus have no access to those properties.

### Modifications to Match the Wikipedia Vocabulary

#### Bock and Cutting (1992).

We identified four subjects or attractors which did not have both their singular and plural form in our vocabulary. Below, we provide one condition (singular subject, singular attractor, PP modifier) of the edited items containing each of those noun phrases, with the noun appearing in the original items shown in parentheses.

(19) The performer (fire-eater) in the carnival show
(20) The inspector (superintendent) of the technical school
(21) The letter (memo) from the junior executive
(22) The lab (laboratory) with the analog computer

In addition, there were 3 words that were not in the Wikipedia training set that were not a part of the critical manipulation, and thus remained as <UNK> tokens during simulations. We provide example sentences containing those words below:

(23) The performer who <UNK> (enlivened) the show
(24) The neural zone around the <UNK> (arcturian) solar system
(25) The traffic jam on the <UNK> (Okemos) street

#### Franck et al. (2002).

All of the words used in the experimental materials were within the Wikipedia vocabulary with one exception, *innkeeper*. We provide a sample sentence of the item with *innkeeper*, and its replacement, *inn*:

(26) The meal for the guest of the inn (inn-keeper)

#### Haskell and MacDonald (2005).

A sample sentence for each item with changes is listed below:

(27) Ask Ronnie if the pearl (ruby) or the diamonds
(28) Do you remember if the table (dresser) or the beds
(29) Did Naomi say whether the shelf (bookshelf) or the beds
(30) Marcus will tell you whether the pitcher or the pots (teapots)
(31) Do you remember if the cocktail (martini) or the beers
(32) Find out whether the shovel or the buckets (rakes)

No <UNK> tokens remained after these changes.

#### Humphreys and Bock (2005).

No words in the Humphreys and Bock (2005) experimental materials were not in the Wikipedia vocabulary, and thus no modifications were made to the items.

#### Parker and An (2018).

One word critical to the manipulation, *stewardess*, was replaced as follows:

(33) The woman (stewardess) who sat the passengers certainly was very pleased with the long flight.

The adverb *unsurprisingly*, though not critical to the manipulation, was also not in the vocabulary. An example sentence with it replaced with an <UNK> token is provided below:

(34) The waitress who sat near the girl <UNK> (unsurprisingly) was unhappy about all the noise.

#### Wagers et al. (2009).

Two words, one critical to the manipulation and one not, were not in the Wikipedia vocabulary. An example item with both words is shown below:

(35) The vendor who the host (hostess) suggests to their friends are excellent but <UNK> (outrageously) expensive.

### WSJ Corpus Items

#### Bock and Cutting (1992).

1. The new tape from the popular rock artist
2. The newspaper from the British government agency
3. The performer in the carnival show
4. The bright light in Doctor Smith’s examination room
5. The security force at the giant manufacturing plant
6. The interview of the famous television host
7. The popular leader of the left dissident group
8. The teacher for the chemistry student
9. The inspector of the technical school
10. The letter from the junior executive
11. The neutral area around the <UNK> solar system
12. The traffic block on the <UNK> street
13. The office of the certified employee
14. The rebel in the dangerous conflict
15. The actor in the blockbuster film
16. The consultant for the growing firm
17. The teaching aide for the science lab
18. The employee with the diplomat’s message
19. The star of the <UNK> production
20. The corporation with the banking monopoly
21. The picture of the prominent politician
22. The writer of the modern book
23. The teacher with the special education certificate
24. The member at the union meeting
25. The director of the new motion picture
26. The candidate for the corporate promotion
27. The editor of the history book
28. The lab with the old computer
29. The activist at the political rally
30. The student in the Spanish class
31. The Peace Corps member in the African town
32. The leader of the Roman city state

#### Franck et al. (2002).

1. The ad from the office of the real estate agent
2. The announcement by the director of the foundation
3. The article by the writer for the magazine
4. The author of the speech about the city
5. The computer with the program for the experiment
6. The contract for the actor in the film
7. The dog on the path around the lake
8. The friend of the editor of the magazine
9. The gift for the daughter of the tourist
10. The helicopter for the flight over the hill
11. The lesson about the government of the country
12. The letter from the friend of my brother
13. The book by the developer of the machine
14. The chair on the deck of the ship
15. The gift for the guest of the hotel
16. The museum with the picture of the artist
17. The design for the engine of the plane
18. The payment for the service to the school
19. The photo of the girl with the baby
20. The post in the support for the platform
21. The prescription by the doctor from the clinic
22. The producer of the movie about the artist
23. The publisher of the book about the king
24. The setting for the movie about the scientist
25. The sign in the garden near the mansion
26. The switch for the light in the room
27. The message to the friend of the politician
28. The threat to the president of the company
29. The tour of the garden near the park
30. The train to the city on the lake
31. The truck on the bridge over the stream
32. The discussion about the topic of the paper

#### Haskell and MacDonald (2005).

1. Can you ask <UNK> if the kids or the adult
2. Do you know if the mice or the monitor
3. Do you think the soybeans or the apple
4. Have you heard whether the teachers or the principal
5. How do I know if the shelves or the floor
6. I <UNK> tell whether the doctors or the professional
7. Do the <UNK> say if the stores or the restaurant
8. We need to know if the potatoes or the grain
9. I want to know if the sheets or the color
10. I need to know if the tables or the chair
11. Maria probably knows if the photos or the painting
12. It didn’t matter to me if the magazines or the book
13. It is hard to tell whether the steelmakers or the engineer
14. Ask <UNK> if the metals or the diamond
15. I wonder if the plants or the fly
16. It doesn’t really matter whether the contractors or the bank
17. Can you tell me whether the swings or the court
18. Do you think the windows or the wall
19. Do you remember if the doors or the carpet
20. Did <UNK> say whether the book shelves or the desk
21. Can you ask the guide if the pencils or the gun
22. Did <UNK> say whether the lights or the plant
23. Can you tell me if the TVs or the phone call
24. Can you tell me whether the boxes or the can
25. The book must say whether the trails or the river bank
26. Would you say the fax machines or the printer
27. Ask the doctor whether the passengers or the driver
28. Marcus will tell you whether the pipelines or the road
29. Do you remember if the waters or the beer
30. Ask the boss if the cases or the box
31. <UNK> confused about whether the pictures or the prize
32. Do you think the lights or the sign
33. Find out whether the prices or the tax
34. Did you think the teams or the expert
35. Can you find out if the barrels or the package
36. Do you know whether the phones or the camera
37. The board wants to know if the theaters or the coffee shop
38. <UNK> must know whether the book stores or the restaurant
39. Can you tell me whether the brokers or the salesman
40. Tell me whether the boards or the president
